# Effects of Fibrinogen and Thrombin Concentrations on Clot Structure and Internal Clot Lysis Time

**DOI:** 10.3390/biom16101471

**Published:** 2026-10-09

**Authors:** Arezoo Nameny, Austin DeSmet, Can Cai, Zezhong Zhang, Stephen R. Baker, Keith Bonin, Nathan E. Hudson, Brittany E. Bannish, Martin Guthold

**Affiliations:** 1 Department of Physics, Wake Forest University, Winston-Salem, NC 27109, USA; 2 Department of Biology, Wake Forest University, Winston-Salem, NC 27109, USA; 3 Department of Physics, East Carolina University, Greenville, NC 27885, USA; 4 Department of Mathematics and Statistics, University of Central Oklahoma, Edmond, OK 73034, USA

**Keywords:** plasma clot structure, internal lysis, fibrinogen, thrombin

## Abstract

Background: In the final steps of the coagulation cascade, thrombin converts fibrinogen to fibrin, which polymerizes into a fibrin network that stabilizes blood clots. The concentration of these two proteins can affect network structure. The fibrin network is dissolved by plasmin, by cleaving fibrin peptide chains. In internal fibrinolysis, tissue plasminogen activator (tPA) activates endogenous plasminogen to plasmin, with activation efficiency much enhanced when tPA and plasminogen are bound to fibrin. The effect of varying fibrinogen and thrombin concentrations and the effect of the resulting varying network structure on fibrinolysis are convoluted, with contradictory trends reported in the literature. Objectives: Investigate how fibrinogen and thrombin concentration quantitatively affect fibrin network structure, and how fibrinogen, thrombin concentrations and the resulting, altered network structure affect fibrinolysis, at a given, rate-limiting tPA concentration. Methods: Fibrin clots were generated from diluted, platelet-poor plasma and purified PEAK 1 fibrinogen, at varying fibrinogen and exogenous thrombin concentrations. The effects of varying fibrinogen and thrombin concentrations on network structure were quantified using non-linear power-law equations derived from confocal images. Internal clot lysis time (CLT) was determined using turbidity-based assays as a function of fibrinogen and thrombin concentrations and network structure at fixed, rate-limiting tPA, and therefore fixed plasmin concentration. Results and Conclusions: Network structural features (fiber density, pore size) and CLT fit well to empirical equations of the form *k*([*Fgn*]*^α^*× [*Thr*]*^β^*). Generally, increasing fibrinogen resulted in higher fiber density and thicker fibers, whereas increasing thrombin resulted in higher fiber density and thinner fibers. Internal CLT showed strong dependence on fibrinogen, and negligible dependence on thrombin. This implies that fibrinogen is the main determinant of internal CLT in these systems, while thrombin and, thus, thrombin-dependent formation kinetics and network structure, per se, have a near-negligible effect.

## 1. Introduction

### 1.1. Fibrin Fiber Formation

**Figure 1:**
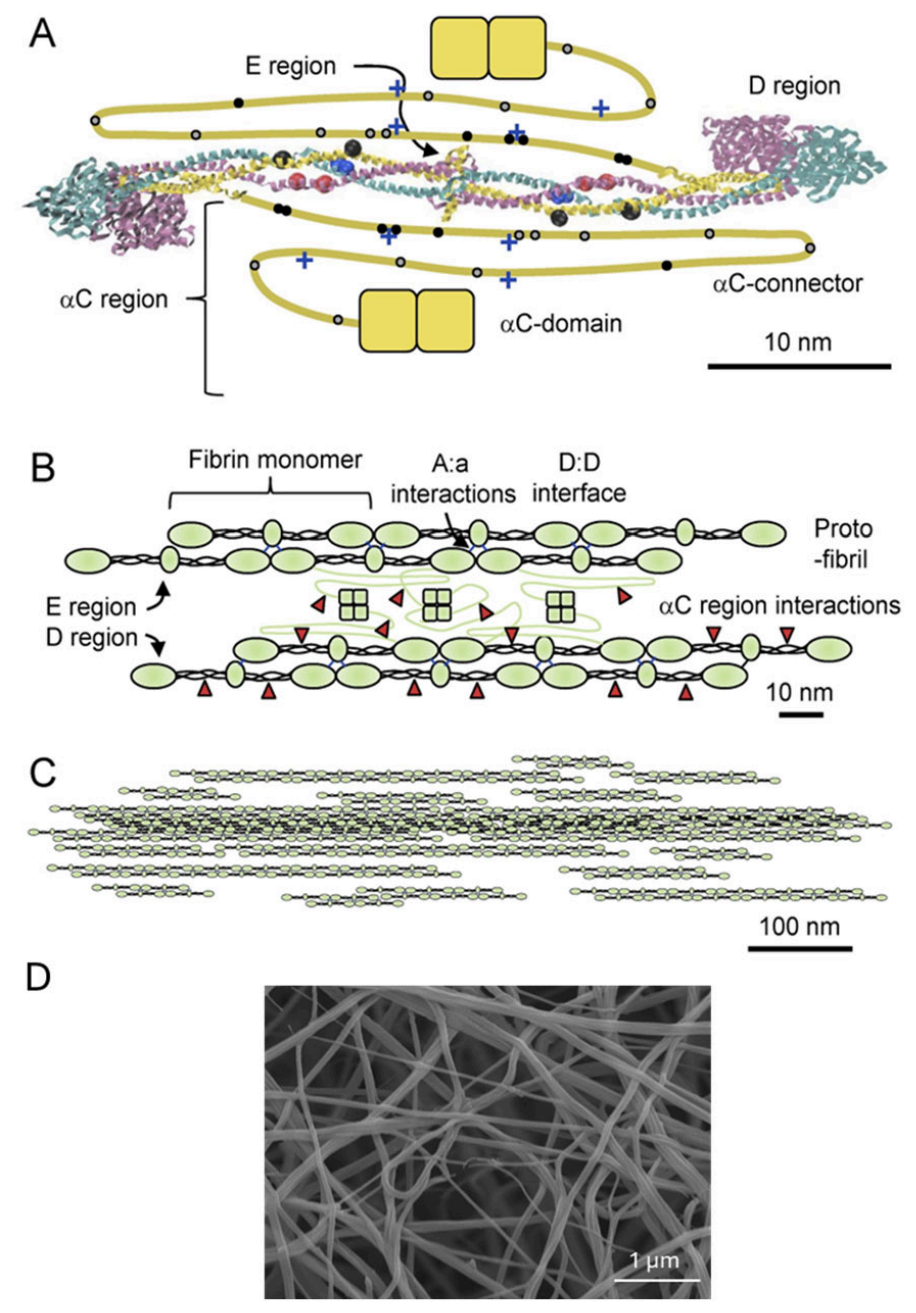
Schematic of fibrin fiber assembly. (**A**) Fibrinogen is composed of two sets of Aα (yellow), Bβ (magenta), and γ (cyan) chains that form a trinodular structure with a central E domain and two outer D domains connected by coiled coils (based on crystal structure [14]). The Aα chains extend into flexible αC regions, which include an unstructured αC-connector and a folded αC-domain (drawn in by hand). Multiple plasmin cleavage sites, indicated by spheres, are located within the coiled coils and αC regions at αLys 81, βLys122, γLys58, and αArg104, βLys133, γLys62. The numerous possible plasmin cleavage sites in the αC-connector are indicated by black dots (lysine), or gray dots (arginine). The blue crosses mark four glutamine residues within the αC-connector that function as donor sites for α-α glutamine–lysine crosslinking. (**B**) Schematic illustration of two double-stranded protofibrils linked through interactions between αC regions [15]. Mature fibrin fibers (~100 nm diameter) contain approximately 100 protofibrils per cross-section connected by an extensive αC network. Selected plasmin cleavage sites are indicated on the lower protofibril by red triangles. (**C**) Longitudinal cross-section of a fibrin fiber showing assembled protofibrils organized into a dense inner core and a less dense outer periphery [16]. (**D**) SEM image of fibrin fiber network formed from human plasma (fibrinogen and thrombin concentration are 0.73 mg/mL and 0.1 U/mL, respectively). (**A**–**C**) are adapted from ref. [17] (original format) and [18] (shown format), with permission.

One of the main structural components of a blood clot is a mesh of fibrin fibers. This fibrin network stabilizes the clot, and traps red blood cells and other blood components, as blood flow is halted. The fibrin network is formed at the end of the coagulation cascade, when thrombin converts soluble fibrinogen to insoluble fibrin by enzymatically cleaving a pair of fibrinopeptides A, and a pair of fibrinopeptides B, from the central E domain of fibrinogen [1]. Removal of these fibrinopeptides exposes amino acid sequences, termed ‘knob A’ and ‘knob B’, which can bind to binding pockets termed ‘hole a’ and ‘hole b’, respectively, in the distal D domains of fibrin [1]. Knob-hole binding between fibrin molecules results in the initial formation of fibrin multimers, half-staggered protofibrils, and fibrin aggregates [2,3]. These initial, smaller fibrin structures then further assemble into a branched network of thicker fibrin fibers [4] (Figure 1).

Human fibrinogen is a 340 kDa plasma glycoprotein composed of two sets of three polypeptide chains (Aα, Bβ, and γ), arranged into a trinodular structure consisting of a central E region and two terminal D regions connected by coiled coils (Figure 1A). Extending from the coiled coils are the flexible αC regions, which are thought to contribute to interactions between protofibrils and stabilization of mature fibrin fibers [5,6]. Mature fibrin fibers contain approximately one hundred protofibrils per cross-section and exhibit a dense core surrounded by a less dense periphery (Figure 1B,C). In addition, activated factor XIII strengthens fibrin fibers through covalent crosslinking between fibrin molecules, thereby increasing clot stiffness and resistance to fibrinolysis [7,8,9,10].

Although the basic thrombin-mediated fibrin activation steps and early fibrin multimer formation are well established, intermediate and later structures of fibrin assembly have not been fully characterized, and the key interactions that control lateral assembly, fiber thickening and branching are also not understood [11,12]. A quantitative analysis of how the two main contributors of fibrin assembly—fibrinogen and thrombin—affect network structure would, therefore, advance our understanding of fibrin network formation [13].

### 1.2. Network Structure

Fibrin network structure is typically characterized by structural parameters, such as fiber density (e.g., fibers/volume or fibers/area), pore size, branchpoints, and fiber diameter [19]. Network structure can be profoundly altered by several variables, such as the concentration of the building block, fibrinogen, and its enzymatic activator, thrombin, as well as salt concentration and molecules that interact with fibrinogen, fibrin and thrombin [13,19,20]. In general, increasing fibrinogen results in higher fiber density and thicker fibers, while increasing thrombin results in higher fiber density and thinner fibers [13,21,22]. Variations in fibrinogen and thrombin concentrations are physiologically relevant in various scenarios. Fibrinogen concentration in healthy individuals ranges from 2–4 mg/mL (6–12 μM) [12]. However, fibrinogen concentration can decrease to near-zero, e.g., in trauma, which may result in dilutional coagulopathy. In this case, severe blood loss is compensated for by increasing blood volume with fluid (without coagulation factors), essentially resulting in a dilution of blood and all plasma components [23]. Fibrinogen levels can increase severalfold in inflammation, disease or obesity, often resulting in thrombotic complications. Thrombin concentration is highly dynamic and can vary by over two orders of magnitude, from 1 nM to 500 nM, depending on temporal and spatial coagulation activation [24,25].

Despite the known, important physiological variations in fibrinogen and thrombin concentrations, the relationships between structural parameters and fibrinogen/thrombin concentrations have mostly been described as trendlines or linear regressions [13]. Moreover, these relationships have often been reported using the ratio of fibrinogen and thrombin concentrations ([Fgn]/[Thr]), which results in inconclusive trendlines. This is because the effect of [Fgn] and [Thr] on structural features is likely non-linear and may have opposing effects on some structural parameters (e.g., increasing fibrinogen causes the fiber diameter to increase, while increasing thrombin causes diameter to decrease). Non-linear equations of the form [*Fgn*]*^α^* × [*Thr*]*^β^* (product of power law functions), rather than the simple linear regressions or ratios of the two, may, therefore, be a better choice to characterize structural parameters as a function of fibrinogen and thrombin concentrations. Such power law functions are more flexible and provide accurate fits with a low number of fitting parameters. This approach yields quantitative expressions describing the effect of thrombin and fibrinogen on structure [26]. Additionally, such equations would lay the baseline for concentration-structure relationships, which will enable accurate modeling and testing of the effect of additional extraneous factors on structure against this baseline.

Moreover, having a way to quantitatively control network structure by varying fibrinogen and thrombin concentrations will make it possible to test the effect of these enzyme concentrations, and the effect of the resulting, altered network structure on fibrinolysis.

### 1.3. Lysis and Clot Lysis Time

Blood clots are transient structures by design, as they carry within them the agents of their dissolution. Plasmin, the activated form of plasminogen, breaks down blood clots by transversely cleaving the fibrin fibers that stabilize a clot [27,28]. Plasmin cuts the fibrin peptide chains at numerous sites, mostly in the αC region and across the coiled coils, which ultimately dissolves fibrin into fibrin degradation products (Figure 1) [29]. Plasminogen is activated by tissue plasminogen activator (tPA) under typical physiological conditions. When plasminogen and tPA are both bound to fibrin, tPA-mediated plasminogen activation is at least 1000 × faster compared to when these enzymes are free in solution [27,30,31]. In internal fibrinolysis, endogenous plasminogen and tPA bind to fibrin fibers as they form; plasminogen and tPA are, therefore, incorporated into the clot. In vitro, internal lysis can be mimicked by forming plasma clots in the presence of small, rate-limiting amounts of tPA, which activates plasminogen that is already present in plasma [32,33]. In contrast, in external fibrinolysis, tPA is added externally to the outside of the clot, after the clot has been formed. External fibrinolysis is important for clinical treatment of pathological clots but is not investigated in the current work.

The effect of clot structure on internal lysis has been examined previously [30,34,35], suggesting that internal clot lysis time may depend on structure; specifically, that clots consisting of more, thinner fibers lyse slower than clots with fewer, thicker fibers.

However, experimental conditions varied widely among these studies, making it difficult to distinguish the effects of fibrin network structure from those of fibrinogen and thrombin concentrations, which themselves influence clot structure. Therefore, it is possible that internal clot lysis time (CLT) depends primarily on fibrinogen concentration rather than on fibrin network structure per se. In this case, network structure would not be an independent determinant of internal CLT, but rather a secondary consequence of changes in fibrinogen concentration.

Prior studies on internal clot lysis have yielded mixed conclusions regarding the role of fibrin network structure. Turbidimetry, confocal microscopy, and qualitative assays suggest that, while individual fiber thickness can affect fibrin cleavage rates, network structure alone does not consistently determine overall clot lysis time when fibrinogen concentration is controlled [30,34,35,36]. Although case–control studies consistently associate prolonged clot lysis times with thrombotic risk, findings regarding fibrin network structure are contradictory, with some studies reporting thicker fibers and others reporting thinner, denser fibers in thrombotic conditions [37,38]. These studies are limited by potential confounding variables and their inability to establish a causal relationship between fibrin network structure and clot lysis time.

To isolate these factors, the current work systematically varies fibrinogen and thrombin concentrations and quantifies their effects on both fibrin network structure and internal CLT. The structural measurements provide the necessary basis for determining whether apparent associations between network structure and CLT reflect a direct dependence of CLT on structure or instead arise from their shared dependence on fibrinogen or thrombin concentration. By independently varying fibrinogen and thrombin, we therefore sought to distinguish the effects of clot composition from those of the resulting network structure on internal fibrinolysis. Experiments were performed in both a dilutional platelet-poor plasma system and a purified fibrinogen system under controlled, rate-limiting tPA conditions.

## 2. Materials and Methods

### 2.1. Clot Formation

Pooled normal human plasma (George King Bio-Medical, Overland Park, KS, USA, Product No. 0020, LOT 7716; stock concentration, 3.25 mg/mL) was aliquoted and stored at −80 °C until use. This is pooled, well-characterized, platelet-poor plasma collected by plasmapheresis from over 30 healthy, non-smoker donors. Prior to experiments, aliquots were thawed at 37 °C for 10 min. Fluorescently labeled fibrinogen (Alexa Fluor™ 488 conjugate (AF488 fibrinogen), Thermo Fisher Scientific, Waltham, MA, USA) was added to plasma at 6.5% of the total fibrinogen concentration. All fluorescently labeled samples, therefore, contained the same final concentration of 6.5% AF488 fibrinogen. The highest possible final fibrinogen concentration was 2.9 mg/mL. To achieve lower fibrinogen concentrations, plasma was serially diluted by a factor of 2 with Tris-buffered saline (TBS; 20 mM Tris, 150 mM NaCl, pH 7.4) to get final fibrinogen concentrations of 2.9 (starting), 1.45, 0.73, and 0.36 mg/mL. Clotting was activated by adding activation mixture containing Tris-buffer saline, CaCl_2_, and thrombin (Enzyme Research Laboratories, South Bend, IN, USA) to final thrombin concentrations of 0.05, 0.1, 0.2 and 1.0 NIH units/mL (U/mL; 1.0 U/mL = 10 nM) and 20 mM CaCl_2_. 30 μL of the clotting reactions were assembled in microtubes and then quickly loaded into microscopy channel slides (ibidi μ-Slide VI 0.4, Thermo Fisher Scientific) for confocal microscopy imaging. Samples were protected from light and incubated at room temperature for 2 h before imaging. Three independently prepared samples, each containing four clots, were analyzed for each fibrinogen/thrombin condition set. Three confocal images were acquired from each clot, resulting in 36 images in total per condition set.

Clots formed from purified fibrinogen (PEAK 1 fibrinogen, Enzyme Research Laboratories) were prepared and analyzed using the same protocol described above. Detailed results on the network structure of these clots, as a function of fibrinogen and thrombin, have been reported previously by our group [26], and the corresponding data are shown here for comparison.

### 2.2. Thrombin Activity

Thrombin activity (for 1 U/mL, 0.1 U/mL and 0 added thrombin (baseline)) in plasma was determined using the Thrombin Activity Assay Kit (Colorimetric; Abcam, Cambridge, UK; Cat. No. ab234620-1001), which enables colorimetric detection of thrombin activity by measuring the absorption of the cleaved substrate at 405 nm in various sample types, including human plasma (Supplement S1). Two independent samples were analyzed for each thrombin concentration.

### 2.3. Confocal Microscopy and Image Analysis

Confocal imaging was performed on a laser-scanning confocal microscope (Zeiss LSM 710, Oberkochen, Germany) using a 40 × oil immersion objective (NA 1.3) and a 488 nm excitation wavelength. For fiber density and bubble/pore diameter, 36 confocal images were analyzed per condition. Image stacks covered an x-y area of 212 μm × 212 μm with a z-stack depth of 15 μm, acquired in 33 slices at 0.47 μm intervals. Images were collected at 8-bit resolution. The master gain was adjusted to visualize fibers while minimizing background noise.

### 2.4. Fiber Density

**Figure 2:**
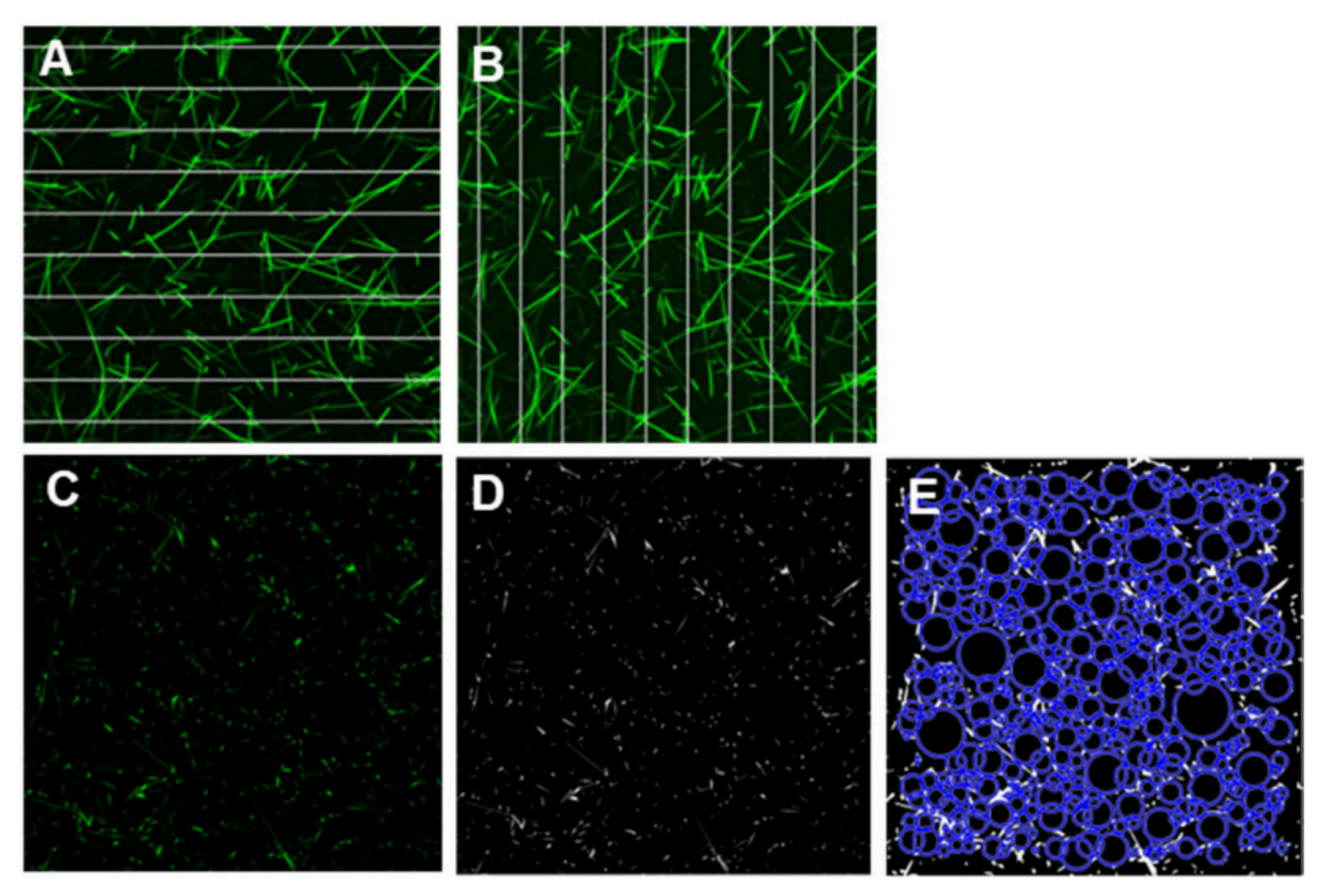
Illustration of the methods used to determine fiber density and bubble/pore diameter. (**A**) Maximum-intensity projection image with horizontal and (**B**) vertical equidistant lines superimposed for fiber density analysis. (**C**) Representative fibrin fiber image slice, (**D**) corresponding binarized image, (**E**) resulting bubble/pore analysis output [39,40].

Image stacks were converted into 2D maximum-intensity projections in ImageJ (version 1.54f), followed by binarization. Twenty horizontal and twenty vertical equidistant lines were superimposed on each image (Figure 2A,B), and the number of fibers intersecting each line was counted (script, see Supplement S7). Fiber density was calculated as the mean number of crossings per 100 µm line length. It should be noted that the image has a depth of 15 µm; therefore, each line is 15 µm deep. Thus, the proper unit for fiber density is fibers/1500 µm^2^. A depth of 15 µm gave images with a good number of fibers, and little background fluorescence.

### 2.5. Bubble Analysis

Image slices were exported to multi-TIFF format and binarized in MATLAB (R2023b, Update 5; MathWorks, Natick, MA, USA) using a threshold of 3× the average image intensity. A custom script (Supplement S8) fits circles (“bubbles”) into fiber-free regions on each slice. Bubbles overlapping with fibers, other larger bubbles, or image boundaries were excluded. The diameters of the remaining bubbles were recorded as a quantitative measure of local pore size, with larger bubble diameters corresponding to larger inter-fiber spaces and greater network porosity (Figure 2C–E) [39,40]. Hereafter, this pore-size metric is referred to as “bubble diameter.”

### 2.6. SEM Analysis

Plasma clots were prepared for SEM imaging, and fiber diameter was analyzed as described in [26,41]. Plasma clots were processed using a standardized wash–fix–dehydrate–dry protocol described previously [41]. After polymerization, clots formed in Eppendorf tube lids were washed three times with sodium cacodylate buffer (50 mM sodium cacodylate, 100 mM NaCl) and fixed by immersion in 2 mL of 2% glutaraldehyde prepared in the same buffer for 6 h. Following fixation, samples were washed three more times with sodium cacodylate buffer, dehydrated through a graded ethanol series (30–100%), and dried using hexamethyldisilazane (HMDS). The dried samples were then sputter-coated with gold for 45 s using a Cressington Model 108 coater (Watford, UK). SEM Imaging was performed using a Zeiss Gemini 300 SEM (Oberkochen, Germany) operated under high vacuum at 30,000× magnification. Two independent clot preparations (biological replicates) were analyzed for each condition. Eight micrographs were collected from each replicate, resulting in a total of 16 images per condition. Images were acquired from widely separated, non-overlapping regions across each clot at a fixed magnification of 30,000×, while avoiding the film region, as previously described [41].

### 2.7. Internal Clot Lysis Time

Clot formation and lysis were assessed by turbidity in a SpectraMax Microplate Reader (San Jose, CA, USA) at 405 nm every 7 s for 3 h in 96 well Microplate (Corning flat clear-bottom white polystyrene, TC-treated). The final reaction volume in each well was 100 μL. Clots were prepared using nine fibrinogen/thrombin concentration combinations: fibrinogen at 0.73, 1.45, or 2.9 mg/mL, and thrombin at 0.1, 0.2, or 1.0 U/mL. Before initiating clotting with the activation mixture, human tPA (Sigma-Aldrich (St. Louis, MO, USA), 70 ng/mL, final concentration), was added to the fibrinogen solution. Three independently prepared clots were analyzed for each fibrinogen/thrombin condition. Lysis time was defined as the time interval between 50% maximum absorbance in clot formation and clot dissolution, as prescribed in Pieters et al. [42].

The same procedure was followed for purified fibrinogen samples, adding 10 ng/mL tPA and 2 µM human plasminogen (Enzyme Research Laboratories, Cat# HPg 2001) to the reactions. Three independently prepared clots were analyzed for each fibrinogen/thrombin condition. Additional experimental details are provided in Supplement S2.

### 2.8. Plasmin Activity Assay

As a supporting assessment of plasmin activity under the plasma experimental conditions with 70 ng/mL tPA, a plasmin activity assay was performed with one replicate per condition. Experimental details and results are provided in Supplement S3.

### 2.9. Statistical Analysis

All statistical analyses were conducted in R version 4.5.1 (open source) using the base “stats” package. The relationship between each response variable and the two experimental factors (fibrinogen and thrombin concentrations) was modeled using a power-function of the form *Y* = *k*([*Fgn*]*^α^* × [*Thr*]*^β^*) where *Y* represents either fiber density or bubble/pore diameter, and *k*, *α*, and *β* are model parameters. Parameters were estimated by non-linear least squares, minimizing the sum of squared differences between the observed and predicted responses. Standard errors were obtained from the covariance matrix of the fitted model. Two-sided *t*-tests were used to assess whether each parameter estimate differed significantly from zero, and corresponding *p*-values were reported. The units used in the fitting routines were mg/mL for [Fgn] and U/mL for [Thr]. Independently prepared samples were treated as the experimental units in the statistical analyses; multiple clots and images obtained from the same independent sample were treated as within-sample measurements rather than independent experimental units.

## 3. Results

### 3.1. Clot Structure as a Function of Fibrinogen and Thrombin Concentrations

**Figure 3:**
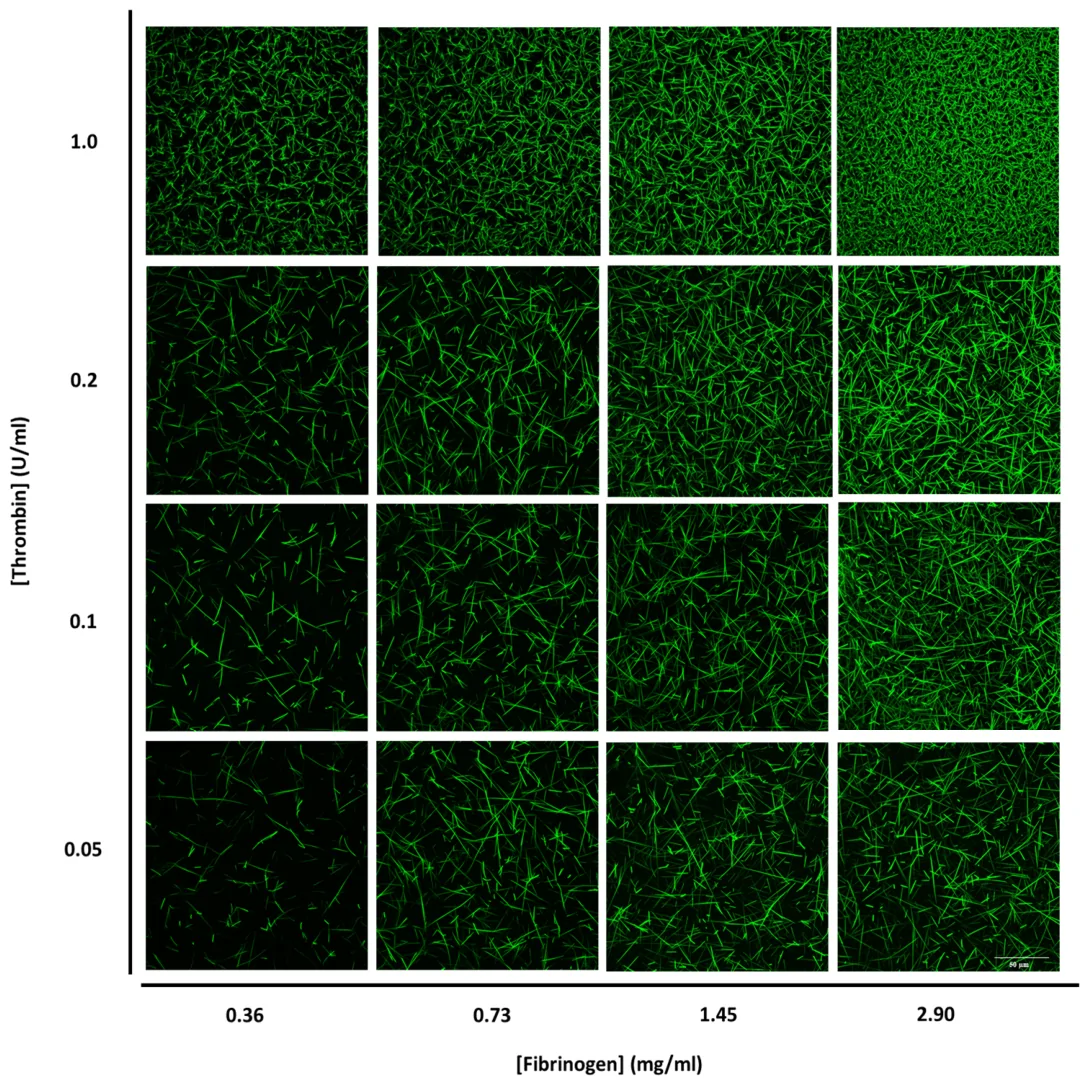
Matrix of maximum-intensity projection confocal images showing plasma clot structures formed at varying fibrinogen (*x*-axis) and added thrombin (*y*-axis) concentrations. Each image represents the fibrin network corresponding to a specific combination of fibrinogen and thrombin levels. Increasing either fibrinogen or added thrombin concentration leads to an increase in fiber density. Scale bar: 50 µm.

**Figure 4:**
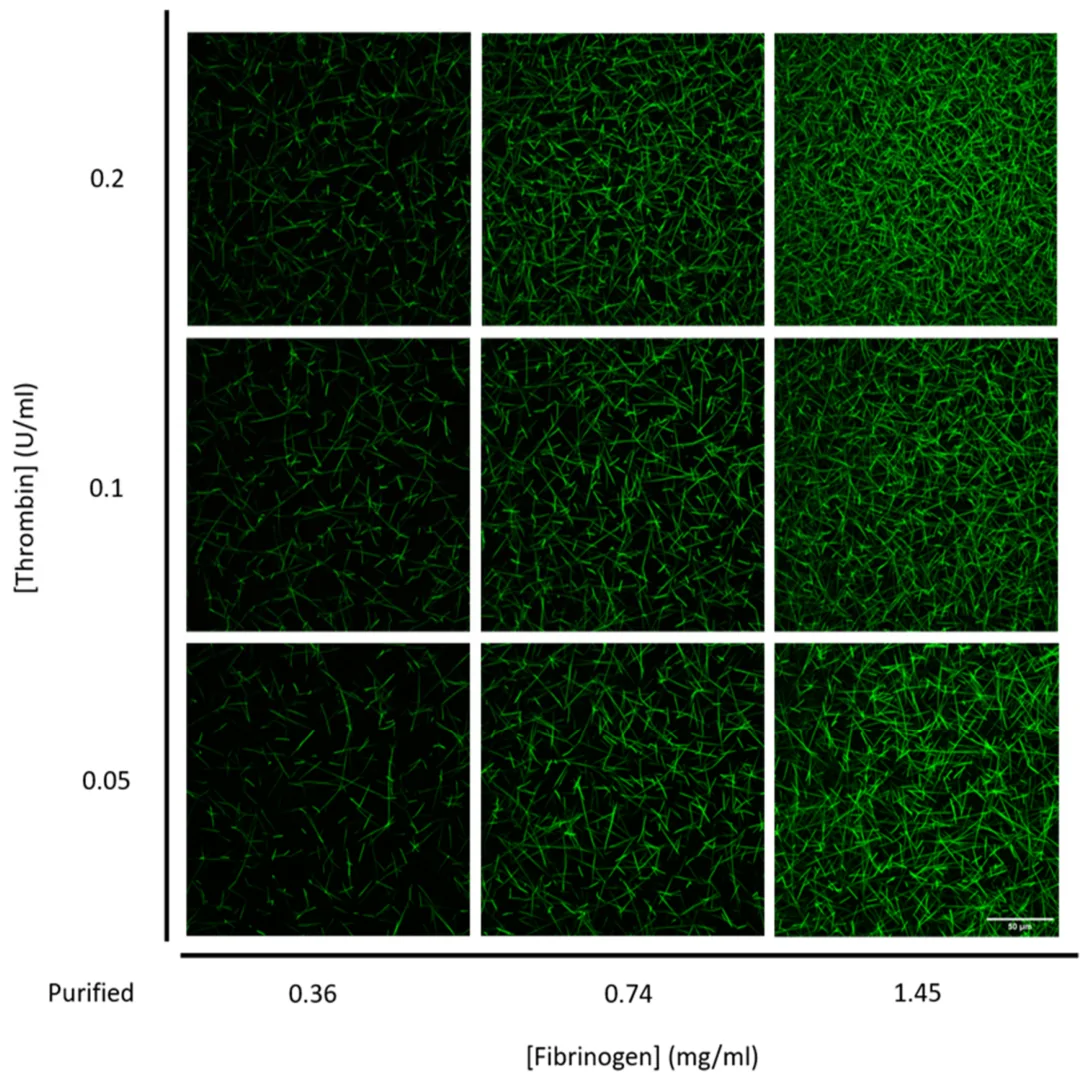
Matrix of representative confocal images showing purified fibrin network structure across fibrinogen (*x*-axis) and thrombin (*y*-axis) concentrations. Each panel corresponds to a specific fibrinogen–thrombin condition. Scale bar: 50 µm. Figure reproduced from ref. [26], with permission.

Confocal microscopy was used to visualize how the fibrin network structure changes with varying fibrinogen and thrombin concentrations. Fibrinogen concentration was adjusted by diluting platelet-poor plasma, and exogenous thrombin was added to these diluted plasmas. Maximum-intensity projection confocal images taken of plasma clots, in which fibrinogen and added thrombin concentration were varied in a 4 × 4 matrix from [Fgn] = 0.36, 0.73, 1.45, 2.9 mg/mL and [Thr] = 0.05, 0.1, 0.2, 1.0 U/mL, are shown in Figure 3. Confocal images of clots formed from purified PEAK 1 fibrinogen at [Fgn] = 0.36, 0.73, and 1.45 mg/mL and [Thr] = 0.05, 0.1, and 0.2 U/mL are shown in Figure 4 (reproduced from ref. [26]). Fibrinogen concentration increases along the *x*-axis, while added thrombin concentration increases along the *y*-axis. In these images, overall fluorescence intensity scales with fibrinogen concentration, and it also depends on imaging parameters, such as gain. The images show a progressive increase in fiber density and a decrease in pore size as both fibrinogen and added thrombin concentrations rise. At lower concentrations, the fibrin network appears sparse, whereas higher concentrations result in dense, highly interconnected networks.

**Figure 5:**
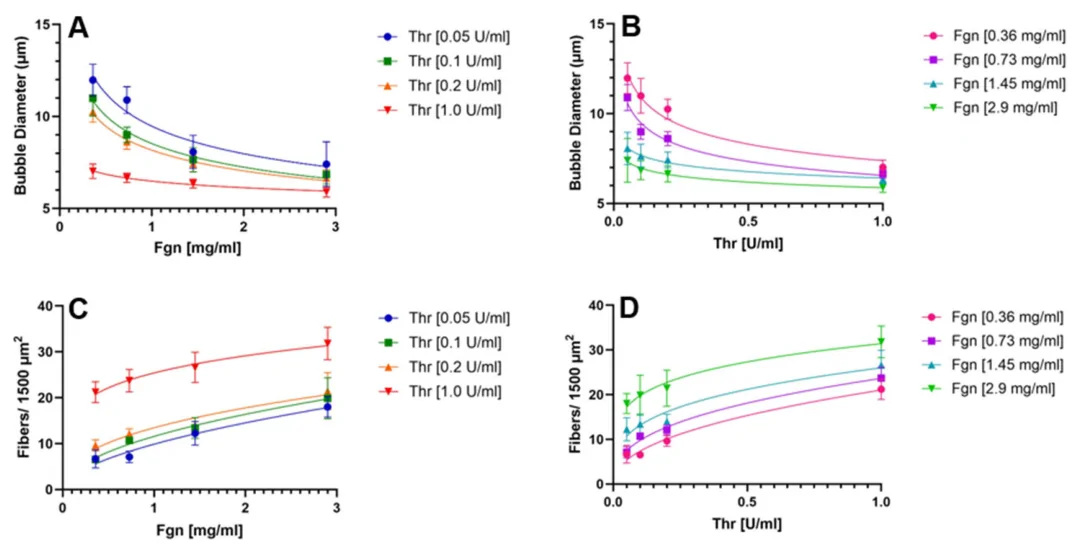
Plasma quantitative analysis of fibrin network structure as a function of fibrinogen and thrombin concentrations and fits by power-law equations (solid lines). (**A**) Bubble/pore diameter versus fibrinogen concentrations, (**B**) bubble/pore diameter versus added thrombin concentrations, (**C**) fiber density versus fibrinogen concentrations, (**D**) fiber density versus added thrombin concentrations. Median values and standard deviations are plotted. Box & whisker plots, the distributions, and data statistics are shown in Supplement S5.

**Figure 6:**
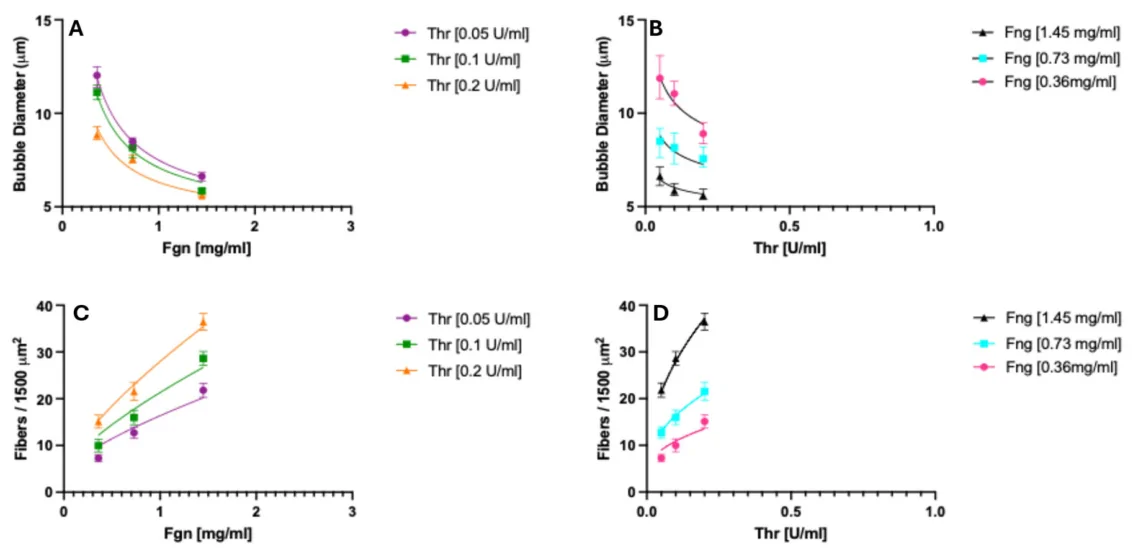
PEAK 1 fibrinogen quantitative analysis of fibrin network structure as a function of fibrinogen and thrombin concentrations and fits by power-law equations (solid lines). (**A**) Bubble/pore diameter versus fibrinogen concentrations, (**B**) bubble/pore diameter versus added thrombin concentrations, (**C**) fiber density versus fibrinogen concentrations, (**D**) fiber density versus added thrombin concentrations. Figure reproduced from ref. [26], with permission.

To quantitatively assess the effect of fibrinogen and added thrombin concentrations on the structure of the fibrin network, we analyzed bubble/pore diameter and fiber density. Plots of these quantities versus concentration reveal a non-linear relationship (Figure 5 and Figure 6). Figure 5 presents the newly acquired plasma structural data, whereas Figure 6 presents the corresponding purified fibrinogen structural data adapted from ref. [26]. To further quantify these non-linear relationships, we fit a power-law model to the plasma data (Table 1); the corresponding power-law analysis for the purified fibrinogen system, adapted from ref. [26], is presented in Table 2. We selected a power law as an empirical fitting equation since it is a simple and versatile mathematical equation with a minimal number of fitting parameters. A power law equation for fibrin structural parameters was derived in our lab by Cai et al. [26]. The results show a clear trend of increasing fiber density with higher fibrinogen and added thrombin concentrations, indicating a denser fibrin network. In contrast, the bubble/pore diameter decreased as the concentrations increased, reflecting the formation of smaller pores within the denser network structure.

For fiber density, all three parameters were highly statistically significant (*p* < 0.001), indicating that both fibrinogen and added thrombin concentrations are positively associated with network structure. The positive exponents imply that fiber density increases with increasing concentrations of both components. For bubble/pore diameter, all parameters were again highly significant (*p* < 0.001). The negative exponents indicate an inverse relationship, demonstrating that increasing fibrinogen or added thrombin concentrations leads to smaller bubble/pore diameters.

### 3.2. Internal Clot Lysis Time and Its Relationship to Fibrin Network Structure

**Figure 7:**
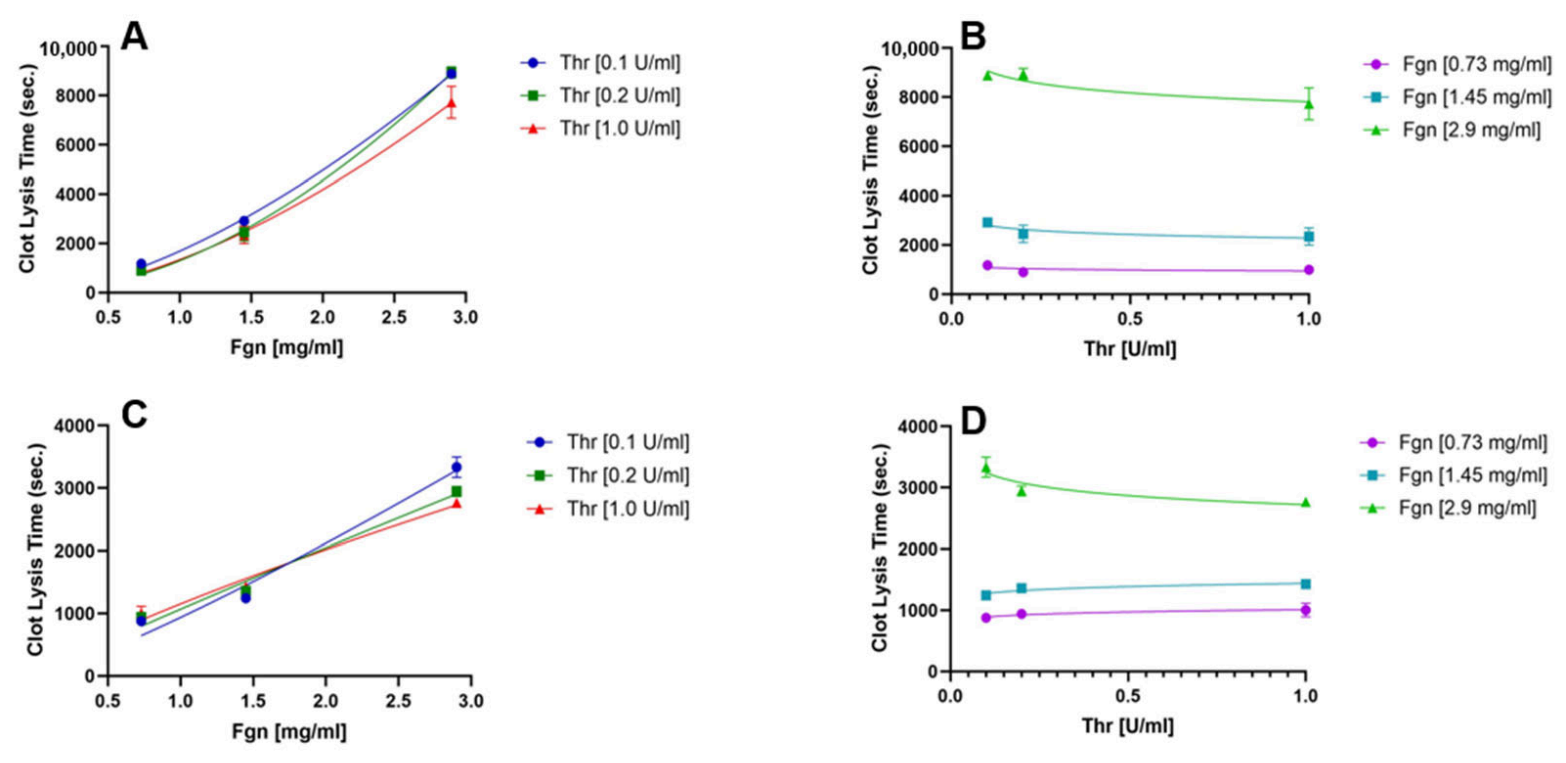
Quantitative analysis of clot lysis time. As a function of fibrinogen concentration in plasma (**A**), in PEAK 1 fibrinogen samples (**C**). As a function of added thrombin concentration in plasma (**B**), in PEAK 1 fibrinogen samples (**D**). See text for fitting using an empirical power-law model of the form *k*([*Fgn*]*^α^* × [*Thr*]*^β^*).

**Figure 8:**
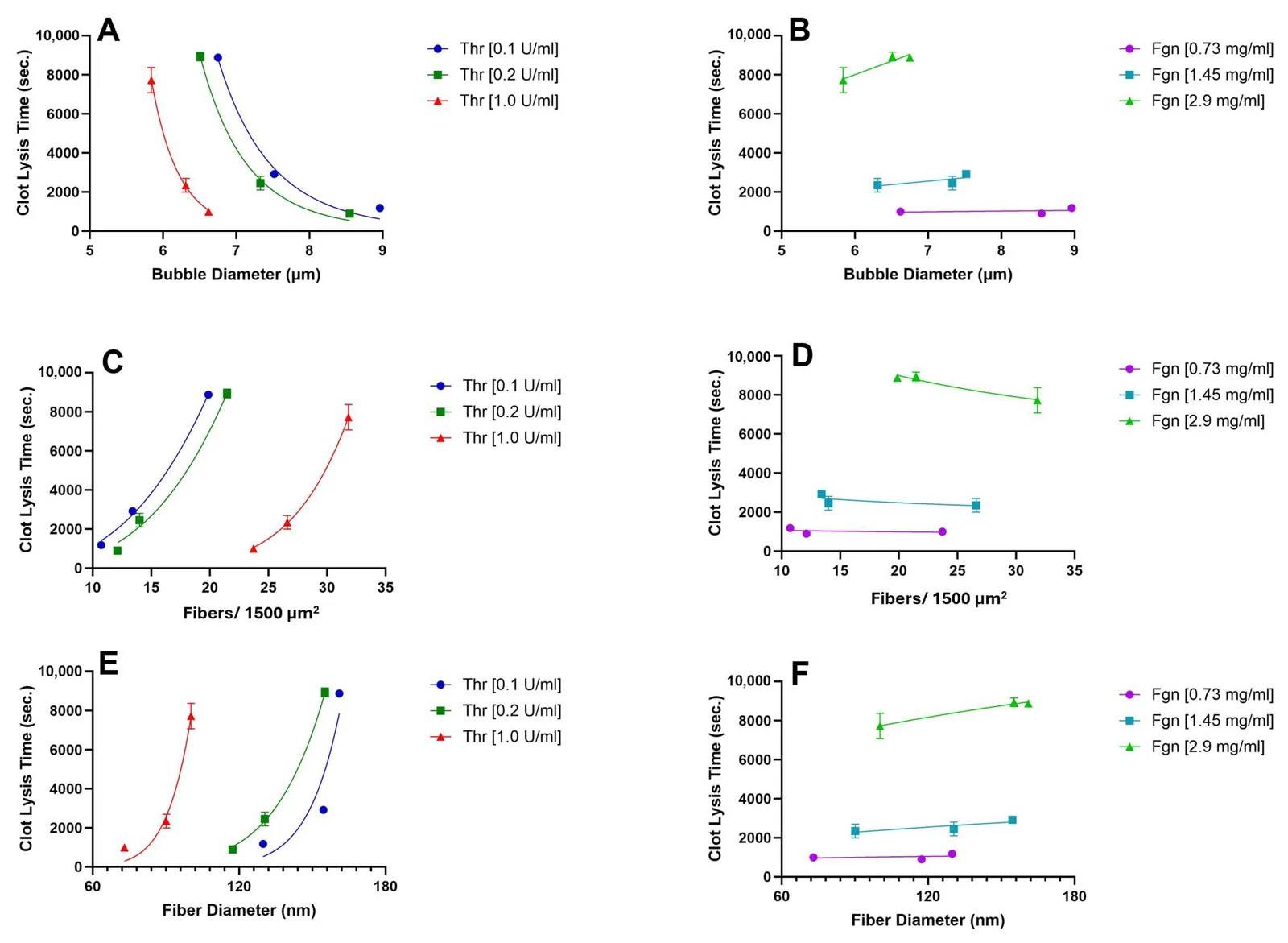
Quantitative analysis of clot lysis time as a function of structural parameters in plasma. Solid lines fit to a power-law equation, CLT = 1.4∙10^3^∙([*Fgn*]^1.54^ × [*Thr*]^−0.07^. (**A**,**C**,**E**) Clot lysis time at three fixed added thrombin concentrations (blue, green, red lines), versus bubble/pore diameter (**A**), versus fiber density (**C**), and versus fiber diameter (**E**). (**B**,**D**,**F**) Clot lysis time at three fixed fibrinogen concentrations (purple, blue, green lines), versus bubble/pore diameter (**B**); versus fiber density (**D**), and versus fiber diameter (**F**). Data points are means of three experiments ± standard deviation. Fiber diameter values were obtained from SEM measurements as described in Section 2.6, reanalysed from an existing dataset form ref. [26].

Plasma clots were formed at various concentrations of fibrinogen (via plasma dilution) and added thrombin in the presence of 70 ng/mL tPA. Similarly, clots formed from purified PEAK 1 fibrinogen at varying fibrinogen and thrombin concentrations were prepared in the presence of 10 ng/mL tPA and 2 μM human plasminogen. In both cases, clot formation and subsequent internal lysis were monitored by absorbance measurements [22,32,42]. In these measurements, absorbance is dominated by light scattering, which depends mainly on fibrinogen concentration, [Fgn], fiber length/mass ratio, μ, and fiber diameter, D [22]. As fibers form, μ and D increase, resulting in increasing absorbance; as the clot is lysed and fibers are dissolved, the absorbance decreases back to baseline. For both plasma and PEAK 1 fibrinogen samples, clot formation was examined in the absence and presence of tPA (Supplement S2). At this concentration, tPA was selected to provide rate-limiting fibrinolytic conditions. For plasma samples, a supporting plasmin activity assay showed comparable plasmin activity across the tested conditions, with no apparent systematic differences (Supplement S3). Subsequently, internal CLT was analyzed as a function of fibrinogen and added thrombin concentrations for both plasma and PEAK 1 samples (Figure 7). For plasma samples, CLT was also analyzed in relation to structural characteristics of the clots, including bubble/pore diameter, fiber density, and fiber diameter (Figure 8). The results show that internal lysis time strongly depends on fibrinogen concentration, regardless of added thrombin concentration (Figure 7). In contrast, clot lysis time has negligible dependence on added thrombin concentration for a given, fixed fibrinogen concentration. These results imply that thrombin-induced kinetics and clot structure have a negligible direct effect on internal clot lysis time. Fitting an empirical power law model of the form *k*([*Fgn*]*^α^* × [*Thr*]*^β^*) to these data yields *CLT* = 1.4∙10^3^∙([*Fgn*]^1.54^ × [*Thr*]^−0.07^) for plasma, and *CLT* = 1.2∙10^3^∙([*Fgn*]^0.82^ × [*Thr*]^0.005^) for PEAK 1 fibrinogen, with CLT in seconds.

The contrasting effects of fibrinogen and thrombin provide a means to distinguish an apparent structure–CLT association from a direct dependence of CLT on network structure. Both fibrinogen and thrombin substantially alter network structure (Figure 3, Figure 4, Figure 5 and Figure 6), whereas fibrinogen has a strong effect on CLT and thrombin has only a weak effect (Figure 7). We therefore next examined CLT as a function of structural parameters while separately considering fibrinogen- and thrombin-dependent structural changes (Figure 8). The structural and CLT measurements were obtained from separate samples and were not paired on a clot-by-clot basis. Instead, the fitted power-law relationships describing each structural parameter as a function of fibrinogen and thrombin concentrations were combined with the corresponding power-law relationship for CLT. Fibrinogen or thrombin concentration was then mathematically eliminated from these equations to derive the relationships between each structural parameter and CLT while separately evaluating fibrinogen- and thrombin-dependent changes. These plots show that clot lysis apparently varies as a function of structural features, namely bubble/pore diameter, fiber density and fiber diameter. However, there is no clear trend. For example, for the same structural feature, e.g., a bubble/pore diameter of about 6.8 μm, or a fiber diameter of 100 nm, the lysis time can vary by a factor of 9, ranging from 1000 s to 9000 s, (Figure 8A,F). Moreover, the same lysis time can be observed for clots with different structural features. Thus, under the experimental conditions investigated, fibrin network structure alone was not sufficient to predict internal clot lysis time.

## 4. Discussion

The main goal of this study was to determine whether internal clot lysis time (CLT) is directly associated with fibrin network structure or whether apparent structure–CLT associations reflect the effects of fibrinogen and thrombin concentrations. The principal finding was that, although both fibrinogen and thrombin substantially altered fibrin network structure, internal CLT depended strongly on fibrinogen concentration but showed weak dependence on added thrombin concentration. Specifically, CLT showed a strong dependence on fibrinogen concentration (power +1.5 in plasma and +0.82 in PEAK 1 fibrinogen), whereas its dependence on added thrombin was practically negligible (power −0.07 in plasma and +0.005 in PEAK 1 fibrinogen). Thus, substantial structural changes induced by thrombin were not accompanied by corresponding changes in internal CLT under the conditions investigated.

Structural characterization was essential to this analysis because it established that both fibrinogen and thrombin produced substantial and quantifiable changes in network structure. Increasing fibrinogen resulted in denser networks with thicker fibers, whereas increasing thrombin resulted in denser networks with thinner fibers. These concentration–structure relationships provided the basis for separately evaluating fibrinogen- and thrombin-dependent structural changes in relation to CLT. Because fibrinogen altered both network structure and CLT, an apparent association between fibrinogen-induced structural changes and CLT does not necessarily establish network structure as an independent determinant of CLT. In contrast, thrombin substantially altered network structure while exhibiting only a weak dependence of CLT on thrombin concentration. Thus, the substantial thrombin-induced structural changes were not accompanied by changes in CLT of comparable magnitude, suggesting that these structural changes do not substantially determine internal CLT under the experimental conditions investigated.

### 4.1. CLT Dependence on Fibrinogen and Not Added Thrombin

Kim et al. [33] showed a linear relationship between internal clot lysis time and fibrinogen concentration, which is in reasonably good agreement with the strong fibrinogen dependence observed in our study. In our experiments, CLT exhibited a superlinear dependence on fibrinogen concentration in the dilutional plasma system (α = 1.54), whereas the dependence in the purified fibrinogen system was closer to linear (α = 0.82). Kim et al. derived this relationship from fundamental kinetic principles and validated it experimentally using a purified fibrinogen system and diluted plasma supplemented with fibrinogen, which differs from our dilutional plasma system (Kim et al. varied fibrinogen, while other plasma factors were constant [33]; our work, fibrinogen and other plasma factors were equally diluted).

Some previous work suggested that internal clot lysis time depends on fibrin network structure. This may be a pseudo-dependence as our work indicates that network structure is not a truly independent variable of internal clot lysis time. Network structure depends on fibrinogen and thrombin concentration (and likely other variables). However, in the dilutional plasma system fibrinogen concentration showed a strong, superlinear dependence of internal clot lysis time, whereas thrombin concentration showed only a weak dependence despite producing substantial changes in fibrin network structure. Similarly, in the purified fibrinogen system, CLT depended strongly on fibrinogen concentration but showed negligible dependence on thrombin concentration, although thrombin substantially altered network structure. Thus, under the experimental conditions investigated, thrombin-induced changes in network structure were not associated with changes in CLT of comparable magnitude. Below, we discuss some of the previous work in this context.

Turbidimetry studies on a purified fibrinogen system [34] investigating internal and external lysis rates suggested that denser clots composed of thinner fibers have a slower internal lysis rate. However, the data on internal lysis used very limited experimental conditions, and a closer inspection and normalization of the data on internal lysis time reveals that the lysis rate is the same for clots with thinner and thicker fibers, at a given fibrinogen concentration.

At times, internal and external lysis time may have been used interchangeably in the literature; however, they need to be clearly distinguished. There is good evidence that clot structure directly influences external lysis, in which tPA is applied to the surface of a clot [37,43,44,45]. In this case, tPA needs to diffuse into the clot. A denser clot might impede this diffusional motion, and, therefore, result in increased external lysis time. For internal lysis, however, tPA is already incorporated into the fibers of a clot and does not need to diffuse into the clot. Therefore, diffusional impediment of tPA motion due to clot structure should not play a role in internal lysis time. However, the internal structure of individual fibrin fibers may affect tPA diffusion and thereby influence fibrinolysis.

In case-control studies [36,37], associations between thrombotic disease, altered clot structure and increased clot lysis time were found. Some of these studies looked at external lysis and, thus, cannot be compared to internal lysis. For internal lysis studies, increased clot lysis time did often, but not consistently, have an association with denser clots consisting of thinner fibers, as some studies showed an association with denser clots consisting of thicker fibers. While case–control studies can establish associations, they have some limitations. In many diseases or conditions, numerous variables are altered, and it is, thus, difficult to isolate a particular variable. Moreover, while associations between observables can be made, definite causation cannot be established. Thus, case-control studies are not inconsistent with our findings.

Our finding that internal clot lysis time depends on fibrinogen concentration aligns with the work by Risman et al. [35] investigating internal lysis of plasma clots using turbidity, confocal microscopy and modeling. Increasing fibrinogen concentration at a constant tPA concentration resulted in increased lysis time, consistent with our findings, whereas maintaining a constant [Fgn]/[tPA] ratio resulted in nearly constant lysis time. Thrombin concentration was not varied in this work; thus, the effect of thrombin-induced structural changes was not investigated.

### 4.2. Power Law Equations for Network Structural Features

For the structural characterization of clots, we focused on fiber density and bubble/pore diameter (pore size) and also used fiber diameter measurements from the same samples from ref. [26]. The error due to limitations in measurement precision are estimated to be around 10%.

We selected empirical power laws of the form *k*([*Fgn*]*^α^* × [*Thr*]*^β^*) for the following reasons. These equations are more versatile than linear relationships, and the exponents can be negative or positive, which allows for increasing or decreasing dependencies. This versatility can be accomplished with few modeling parameters. We also tried exponential fits, but the fits were less accurate. In addition, a derivation of the power law is given in Cai et al. showing that an approximate multiplicative power-law form can arise from a coarse-grained kinetic framework involving thrombin-mediated fibrinogen activation, substrate depletion, and finite-window fiber growth [26]. Although this derivation is not a complete mechanistic model of fibrin assembly, it provides a rationale for why separable power-law-like dependencies can emerge for network structural features over the finite experimental range studied here. Accordingly, these empirical power-law relationships should be interpreted within the concentration ranges investigated and should not be extrapolated to higher fibrinogen or thrombin concentrations, where the assumptions underlying the approximate scaling may no longer apply.

### 4.3. Selection of Fibrinogen and Added Thrombin Concentrations

The pooled normal plasma used in this study had an initial fibrinogen concentration of approximately 3.2 mg/mL. Rather than increasing fibrinogen concentration by supplementing the plasma with exogenous purified fibrinogen, we used a dilutional approach to systematically decrease fibrinogen concentration from this native plasma value. Thus, the lower fibrinogen concentrations investigated (0.36, 0.73, and 1.45 mg/mL) were not intended to represent the normal physiological range, but rather to provide a controlled concentration series extending to markedly reduced fibrinogen levels. Below 0.36 mg/mL, it was difficult to form stable clots, whereas the upper limit was determined by the fibrinogen concentration available in the pooled normal plasma. Added thrombin concentrations were in the range of 0.05 to 1.0 U/mL (0.5 to 10 nM). Physiologically, thrombin concentration is highly dynamic depending on spatial and temporal coagulation status during thrombin generation [24]. Before activation (coagulation initiation), available thrombin is near zero (sub-pM). During coagulation, thrombin concentration can range from 1 nM to 500 nM [24,25]. We were unable to use concentrations higher than 10 nM because clotting was too rapid so that clots formed in the pipette.

These concentration ranges were therefore selected to systematically examine concentration-dependent effects under controlled in vitro conditions and should not be interpreted as reproducing the full range of fibrinogen and thrombin conditions encountered in vivo.

### 4.4. Rate-Limiting tPA and Plasmin Activity

A tPA concentration of 70 ng/mL was selected for the plasma samples to establish controlled, rate-limiting fibrinolytic conditions. This concentration was sufficiently low for tPA-mediated plasminogen activation to remain rate-limiting for fibrinolysis [33] and to permit proper clot formation, as indicated by the development of a plateau phase in the absorbance curves (Supplement S2), while being sufficiently high to ensure complete lysis of all samples within a practical experimental time (≲4 h), before appreciable sample evaporation occurred. The fixed tPA concentration was selected for experimental control and was not intended to reproduce the dynamic concentrations or release of tPA in vivo.

As a supporting assessment of plasmin activity across experimental conditions, plasmin activity was measured using a colorimetric plasmin substrate (Supplement S3). Plasmin activity remained comparable, with no apparent systematic difference across the tested conditions despite variations in fibrinogen and thrombin concentrations. These observations are consistent with the interpretation that the observed differences in clot lysis time were not primarily driven by large differences in plasmin activity.

Nevertheless, the use of a fixed tPA concentration does not reproduce the spatially and temporally dynamic regulation of fibrinolysis in vivo, particularly under pathological conditions such as trauma or sepsis, and the present findings should therefore be interpreted within the context of the controlled in vitro conditions investigated.

The experimental system therefore provided controlled conditions for examining the effects of fibrinogen and added thrombin on fibrin formation and internal fibrinolysis within a plasma background. As an additional validation, results from the dilutional plasma system were compared with those obtained using a purified fibrinogen system. The two systems produced consistent trends: fibrin network structure followed similar power-law relationships [26], and clot lysis time depended strongly on fibrinogen concentration while showing negligible dependence on thrombin concentration (Figure 7).

### 4.5. Added AF488 Fibrinogen, Network Formation and Structure

Previous work showed that fluorescently labeled fibrinogen (AF546) can affect fibrin network formation [16]. Optimally, the fluorophore concentration is used that is low enough to interfere minimally with fiber formation, but high enough to render fibers visible, and label fibers evenly. Since fiber density and fiber diameter both depend on fibrinogen and thrombin concentration, creating a network with fibers that all have equal fluorophore density is impossible in practice. Our approach was to keep the numbers of fluorophores per fiber approximately constant by keeping the percentage of fluorescently labeled fibrinogen constant. We used 6.5% AF488 fibrinogen in all reactions because this amount was sufficiently high to give good visibility of the fibers in all samples, but it was low enough to not interfere with fibrin network formation. We tested higher amounts of AF488 fibrinogen, and it resulted in aggregates and artefacts like star-like shapes. In addition, we performed turbidity experiments with different amounts of fluorescently labeled fibrinogen (0%, 1.5%, 3%, 6%, 12%) and showed that maximum absorbance and clot formation rate were minimally affected by 0–6% AF488 fibrinogen. More significant differences in maximum absorbance were observed at 12% AF488 fibrinogen (Supplement S4). Therefore, adding 6.5% AF488 fibrinogen is a good compromise between having bright, evenly labeled fibers while, at the same time, not affecting network formation and structure.

### 4.6. Dilutional Plasma System

We used a dilutional plasma approach to systematically vary fibrinogen concentration from its native concentration in the pooled plasma without supplementing the system with exogenous purified fibrinogen. A limitation of this approach is that fibrinogen is not varied independently of other plasma components, because clotting factors, inhibitors, and other plasma proteins are simultaneously diluted. However, plasma dilution also has relevance to dilutional conditions that may occur during substantial blood loss and fluid resuscitation [23]. The present system should therefore not be considered a model of dilutional coagulopathy itself, but rather a controlled in vitro system that reproduces one aspect of such conditions, namely the concomitant reduction in fibrinogen and other plasma components. Alternative approaches to varying fibrinogen concentration in a plasma background also have limitations. Plasma could be diluted and subsequently supplemented with exogenous fibrinogen to restore fibrinogen to selected concentrations [33]; however, this would alter the relative concentrations of fibrinogen and other diluted plasma components. Alternatively, fibrinogen-depleted plasma could be supplemented with purified fibrinogen, which would permit more independent control of fibrinogen concentration but would constitute a different experimental system. We therefore selected the dilutional approach for the plasma experiments and complemented it with experiments in a purified fibrinogen system. Importantly, parallel experiments using purified PEAK 1 fibrinogen allowed the effects of fibrinogen and added thrombin concentrations on fibrin network structure and internal CLT to be examined in the absence of other plasma proteins. The two experimental systems showed consistent principal trends: fibrinogen and added thrombin altered fibrin network structure, whereas internal CLT depended strongly on fibrinogen concentration and showed negligible dependence on added thrombin concentration. However, the stronger fibrinogen dependence observed in dilutional plasma (α = 1.54) than in the purified PEAK 1 system (α = 0.82) indicates a quantitative difference between the systems that may reflect differences in experimental conditions, including the use of different tPA concentrations and the concurrent variation in other plasma components during plasma dilution. Nevertheless, both systems represent controlled in vitro models and do not reproduce the full biochemical, cellular, and physiological complexity of clot formation and fibrinolysis in vivo.

### 4.7. Limitations Regarding Thrombin Concentration in Plasma Samples

A potential caveat of working with plasma and adding small amounts of thrombin is that the addition of exogenous thrombin may trigger delayed, further endogenous thrombin generation, resulting in uncertainty of the ultimate total thrombin concentration within the system. At very low added thrombin concentrations (≤0.04 U/mL), additional thrombin may be generated through a factor XI (FXI)-dependent pathway. At higher thrombin concentrations (≥0.8 U/mL), this pathway is effectively bypassed [25]. In our work, however, there is evidence that the added thrombin is the dominant contributor to the thrombin-dependent structural effects. First, we determined thrombin activity using a colorimetric thrombin activity assay (Supplement S1). The results show thrombin activity increases with added thrombin (0.1 U/mL and 1 U/mL) at three different fibrinogen concentrations (0.7, 1.4, 2.5 mg/mL). These thrombin activities are also higher than plasma plus activation mix (no thrombin baseline), which is higher than plasma without activation mix (no thrombin, no re-calcification). Second, the confocal images show that added thrombin has a dose-dependent effect on structure. The added thrombin, therefore, appears to be dominant over the possibly subsequently generated thrombin. Third, in the purified fibrinogen system, in which fibrinogen and thrombin are constant and exactly known, thrombin shows a similar effect on network structure and on clot lysis time. The key findings in the dilutional plasma system and the purified plasma system are consistent. Therefore, while we cannot be certain of the ultimate total thrombin concentration in the dilutional plasma system, these lines of evidence suggest that the added thrombin is dominant and has a dose-dependent effect on structure. The thrombin term in the equations should, therefore, strictly be considered ‘added thrombin’ (and not total thrombin). The key result is that added thrombin has an effect on structure but has a negligible effect on lysis. Nevertheless, the use of fixed concentrations of exogenous thrombin does not reproduce the spatially and temporally dynamic generation of thrombin in vivo. Therefore, the thrombin-dependent relationships reported here should be interpreted within the context of the controlled in vitro conditions investigated.

## 5. Conclusions

We investigated fibrin network structure and internal clot lysis time (CLT) as functions of fibrinogen and added thrombin concentrations under controlled in vitro conditions. Confocal microscopy demonstrated that increasing fibrinogen and added thrombin concentrations produced denser fibrin networks with smaller pores, and these concentration-dependent structural changes were quantitatively described by a power-law model *k*([*Fgn*]*^α^* × [*Thr*]*^β^*). In contrast to their effects on network structure, fibrinogen and added thrombin had markedly different effects on internal fibrinolysis. CLT depended strongly on fibrinogen concentration, whereas added thrombin had only a weak effect over the concentration range investigated. Thus, although both fibrinogen and thrombin substantially altered fibrin network structure, the substantial thrombin-induced structural changes were not accompanied by changes in CLT of comparable magnitude. Under the experimental conditions and fibrinogen/thrombin concentration ranges investigated here, these findings indicate that associations between fibrin network structure and internal CLT do not necessarily establish network structure as an independent determinant of CLT. The present findings establish mechanistic relationships among fibrinogen concentration, added thrombin concentration, fibrin network structure, and internal CLT under controlled in vitro conditions. These experimental systems do not reproduce the full biochemical, cellular, and physiological complexity of clot formation and fibrinolysis in vivo, and the applicability of these relationships to specific thrombotic or bleeding conditions remains to be established. Future studies using patient-derived plasma and experimental systems incorporating additional physiological factors will be necessary to determine whether these relationships are maintained under more physiologically relevant conditions.

## Figures and Tables

**Table 1:** Fitting parameters and statistical results for bubble/pore diameter, fiber density and clot lysis time for the non-linear model *k*([*Fgn*]*^α^* × [*Thr*]*^β^*) in plasma.

		Value	Std. Error	*p*-Value	R^2^
Parameters for Bubble/pore diameter	k	6.4	0.2	8.06 × 10^−13^	0.93
	α	−0.22	0.02	1.55 × 10^−7^	
	β	−0.13	0.02	2.43 × 10^−6^	
Parameters for Fiber density	k	24	1.0	1.65 × 10^−11^	0.92
	α	0.31	0.04	1.11 × 10^−5^	
	β	0.29	0.03	2.25 × 10^−7^	
Parameters for Clot Lysis Time	k	1.4 × 10^3^	1.0	<2 × 10^−16^	0.98
	α	1.5	0.04	<2 × 10^−16^	
	β	−0.07	0.03	0.01	

**Table 2:** Fitting parameters and statistical results for bubble/pore diameter, fiber density and clot lysis time for the non-linear model *k*([*Fgn*]*^α^* × [*Thr*]*^β^*) in PEAK 1 fibrinogen. Data for bubble/pore diameter and fiber density, adapted from ref. [26].

		Value	Std. Error	*p*-Value	R^2^
Parameters for Bubble/pore diameter	k	5.1	1.0	1.2 × 10^−7^	0.98
	α	−0.41	0.02	2.6 × 10^−6^	
	β	−0.14	0.02	0.001	
Parameters for Fiber density	k	57	1.0	3.2 × 10^−6^	0.99
	α	0.73	0.03	3.5 × 10^−6^	
	β	0.43	0.02	0.0005	
Parameters for Clot Lysis Time	k	1.2 × 10^3^	1.0	<2 × 10^−10^	0.93
	α	0.82	0.1	<8 × 10^−5^	
	β	0.005	0.05	0.9	

## Data Availability

The original contributions presented in this study are included in the article/Supplementary Material. Further inquiries can be directed to the corresponding author.

## Supplementary Materials

The following supporting information can be downloaded at: https://www.mdpi.com/article/10.3390/biom16101471/s1, Supplement S1: Thrombin generation assay & baseline thrombin activity in plasma. Supplement S2: Clot lysis time as a function of fibrinogen and thrombin in purified PEAK 1 fibrinogen and plasma systems. Supplement S3: Plasmin Generation for different fibrinogen and thrombin concentrations. Supplement S4: AF488-labeled fibrinogen & network formation and structure. Supplement S5: Box and whisker plots, and distribution corresponding to Figure 5A. Supplement S6: Table S1. Plasma fitting parameters and statistics of bubble diameter (median), and fiber density (mean). Supplement S7: Code for Fiber Count Analysis. Supplement S8: Code for Bubble Analysis (Matlab).
