# Supplementary material for: Effects of Fibrinogen and Thrombin Concentrations on Clot Structure and Internal Clot Lysis Time
Source: Biomolecules. 2026 Oct 9;16(10):1471. doi: 10.3390/biom16101471 (PMC13702216; doi:10.3390/biom16101471)
Supplement: Supplementary file 1 [file biomolecules-16-01471-s001.zip › biomolecules-4557425-supplementary.pdf]

biomolecules

MDPI

Supplementary Information

Table of Contents

S1. *Thrombin generation assay & baseline thrombin activity in plasma*

S2. *Clot lysis time as a function of fibrinogen and thrombin in purified PEAK 1 fibrinogen and plasma systems*

S3. *Plasmin Generation for different fibrinogen and thrombin concentrations*

S4. *AF488-labeled fibrinogen & network formation and structure*

S5. *Box and whisker plots, and distribution corresponding to Fig. 5A, main manuscript*

S6. *Table S1: Plasma fitting parameters and statistics of bubble diameter (median), and fiber density (mean)*

S7. *Code for Fiber Count Analysis*

S8. *Code for Bubble Analysis (Matlab)*

S1. Thrombin generation assay & baseline thrombin activity in plasma.

We determined the baseline thrombin activity in plasma using the Abcam Thrombin Activity Assay Kit (ab234620-1001), according to the manufacturer's instructions. Reactions (100 μL total volume) were assembled in a 96-well plate, with each well containing 10 μL of sample or thrombin standard and 90 μL of thrombin generation assay mixture. Thrombin generation was monitored by measuring absorbance at 405 nm in various sample types, including human plasma. Two independent samples were analyzed for each thrombin condition. The assay measured a baseline thrombin activity of 0.1 U/mL.

In addition, thrombin activity was measured in calcified plasma alone and in calcified plasma supplemented with the lowest and highest added thrombin concentrations used in this study (0.1 U/mL and 1 U/mL). These measurements demonstrated that thrombin activity is very low in non-recalcified plasma, that overall thrombin activity (added thrombin plus generated thrombin) scales with added thrombin, and added thrombin dominates baseline thrombin at 0.1 U/mL and 1 U/mL added thrombin. The domination is more pronounced for higher fibrinogen concentrations.

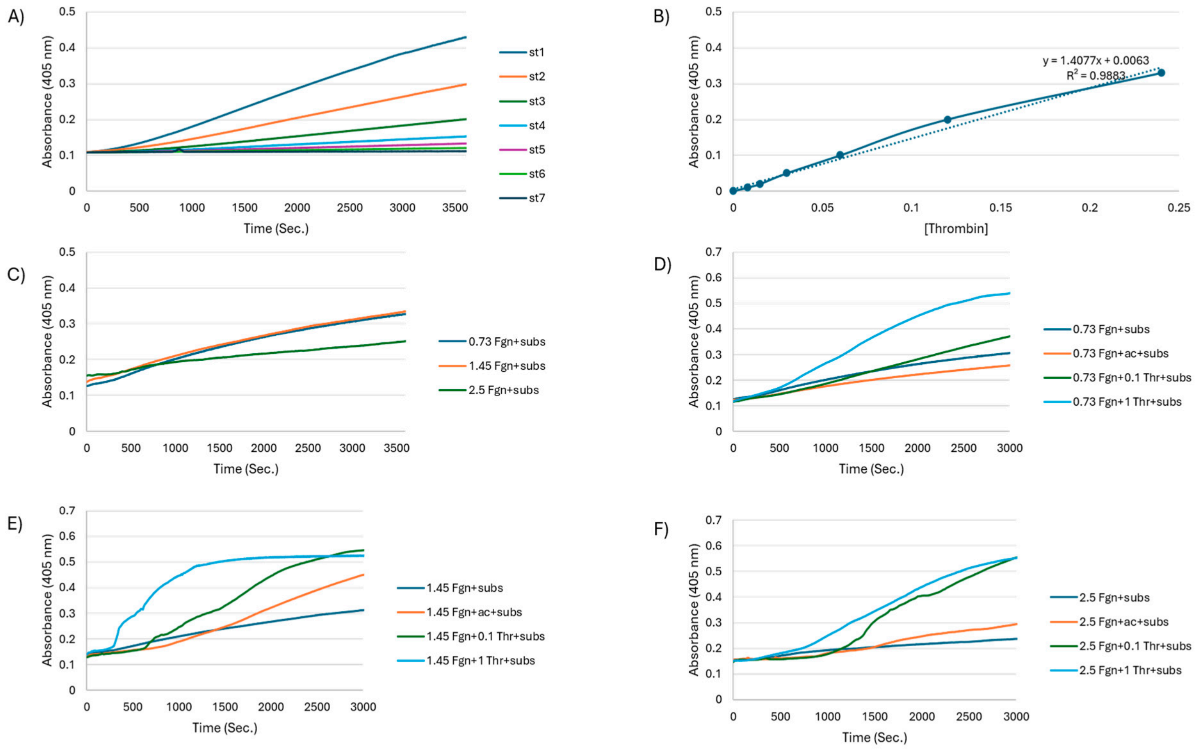

Figure S1. Measurement of thrombin activity in plasma samples using the Abcam Thrombin Activity Assay Kit (ab234620-1001). (A) Standard thrombin calibration curve generated using known thrombin concentration. (B) The linear fit was used to determine thrombin activity in plasma samples. (C) Absorbance vs. time of calcified plasma samples, no thrombin added. (D-F) Comparison of thrombin activity in plasma alone, activated plasma without added thrombin, plasma with the lowest added thrombin concentration (0.1 U/mL), and plasma with the highest added thrombin concentration (1 U/mL).

S2. Clot lysis time as a function of fibrinogen and thrombin in purified PEAK 1 fibrinogen and plasma systems.

We performed CLT experiments using a purified system in which known concentrations of thrombin (0.1, 0.2, 1 U/mL) were added to purified PEAK 1 fibrinogen (0.73, 1.45, 2.9 mg/mL) in the presence of 2 µM human plasminogen (Enzyme Research Laboratories, Cat# HPg 2001) in TBS and 20 mM $CaCl_2$. Reactions were prepared in a 96-well plate with a final volume of 100 µL per well, and clot formation and lysis were monitored by measuring absorbance at 405 nm. (Fig. S2-1). Three independently prepared clots were analyzed for each fibrinogen/thrombin condition.

Fig. S2-2 shows CLT experiments performed in the plasma system using the same fibrinogen and thrombin concentrations as those used in the purified system. Three independently prepared clots were analyzed for each fibrinogen/thrombin condition

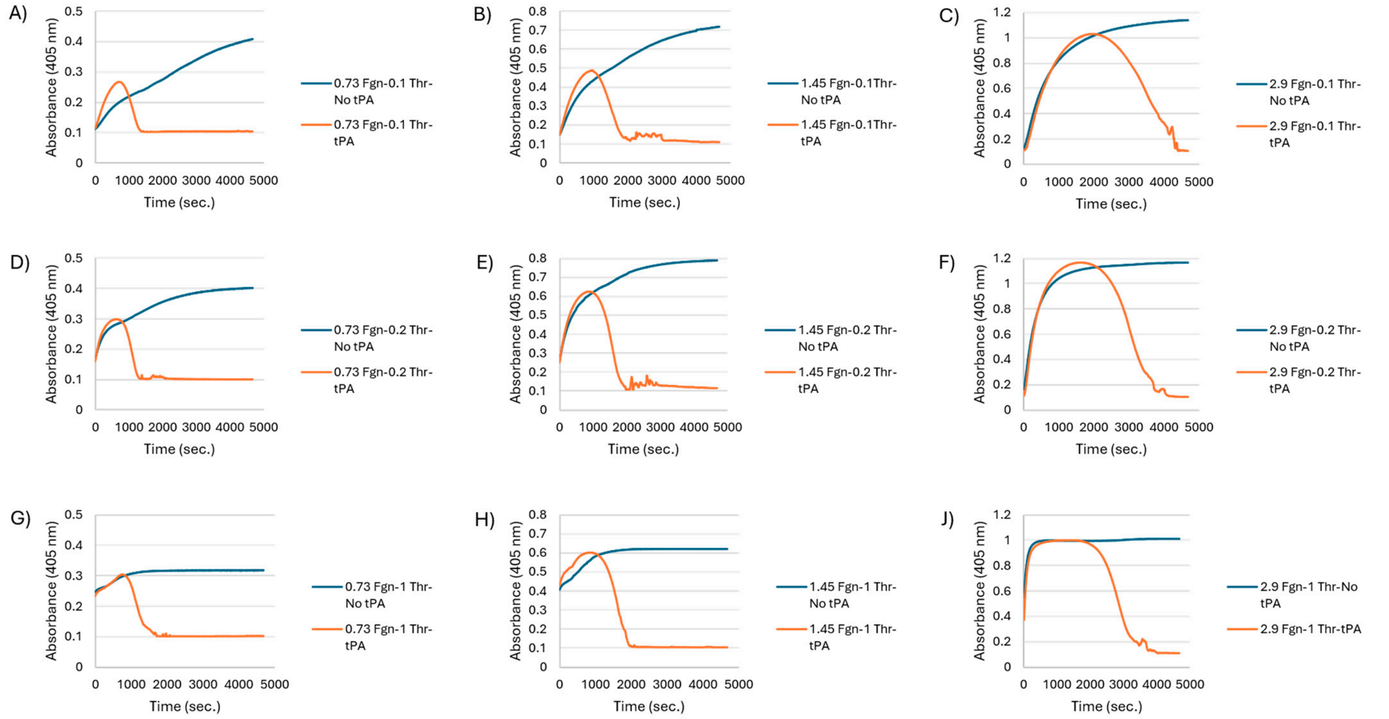

Figure S2. 1: Absorbance measurements of purified PEAK 1 fibrinogen clot formation and lysis at various fibrinogen concentrations, (A–C), (D–F), and (G–J), using 0.1, 0.2, and 1.0 U/ml thrombin, respectively. Curves represent the average of three clot datasets, with and without tPA.

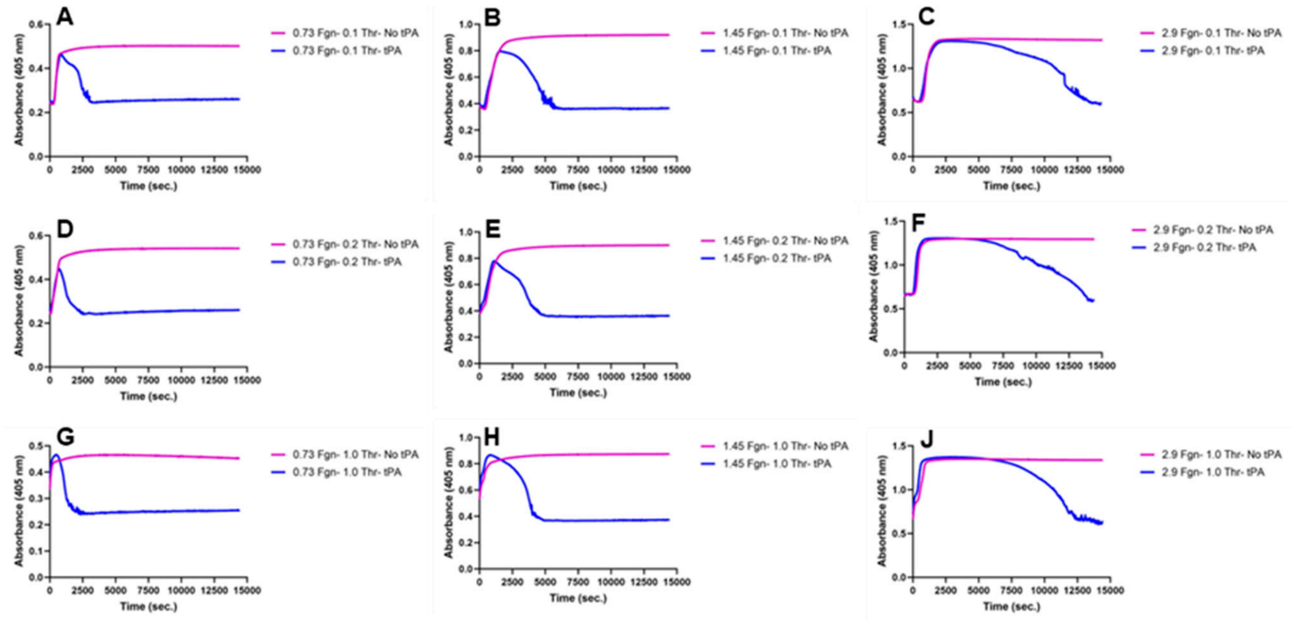

Figure S2. 2: Absorbance measurements of plasma clot formation and lysis at various fibrinogen concentrations, (A–C), (D–F), and (G–J), using 0.1, 0.2, and 1.0 U/ml added thrombin, respectively. Curves represent the average of three clot datasets, with and without tPA.

S3. Plasmin Generation for different fibrinogen and thrombin concentrations.

We performed a plasmin generation assay using colorimetric plasmin substrate, D-Valyl-L-leucyl-L-lysine 4-nitroanilide dihydrochloride (Product# V0088, Sigma-Aldrich). Reactions were prepared in a 96-well plate with a final volume of 100 µL per well and

contained final concentrations of fibrinogen (0.73, 1.45, or 2.4 mg/mL), thrombin (0.1, 0.2, or 1 U/mL), and plasmin substrate (1 mM) in TBS with 20 mM $CaCl_2$. This substrate is specifically cleaved by plasmin, and upon cleavage this substrate absorbs at 405 nm, which can be detected in a plate reader. A fibrinogen concentration of 2.4 mg/mL, rather than 2.9 mg/mL used in the corresponding clot-lysis experiments, was required to accommodate the additional volume of the colorimetric substrate while maintaining the same sample volume and final reaction volume. One replicate was performed for each condition; therefore, variability between independent replicates could not be assessed.

Fig. S3 shows the raw absorbance data for all used fibrinogen and thrombin concentrations. The experiments were performed with and without added tPA (70 ng/mL), and with and without added colorimetric substrate. The control experiments without the colorimetric substrate were performed because the fibrin network also scatters light at 405 nm (which manifests as turbidity), and these control experiments allowed a separation of the turbidity contribution and the substrate absorption contribution of 405 nm absorbance signal.

In the experiments without substrate 'regular' turbidity curves of fibrin network formation and clot lysis are observed. When the plasmin substrate is added to the reaction, the absorbance signal increases significantly, which is due to the absorption of the colorimetric substrate upon cleavage.

The absorbance versus time curves show three phases. 1) A turbidity signal due to fibrin network formation and lysis. 2) In reactions that included the colorimetric plasmin substrate, a strong increase in absorbance due to cleaved substrate absorption. 3) A plateau phase in absorption as the colorimetric substrate is exhausted.

The maximum slope of the absorption signal occurs when the substrate is in excess and plasmin is saturated with substrate. To determine the absorption signal, we subtract the turbidity signal (with no substrate) from the total absorbance signal.

The slope of this absorption signal was then determined for all fibrinogen and thrombin concentrations with and without tPA (Fig. S3I). In this experiment, reactions containing tPA showed substantially higher slopes than reactions without tPA. Among the different fibrinogen/thrombin conditions containing tPA, the measured slopes were comparable, with no apparent systematic difference across the tested conditions.

https://doi.org/10.3390/biom16101471

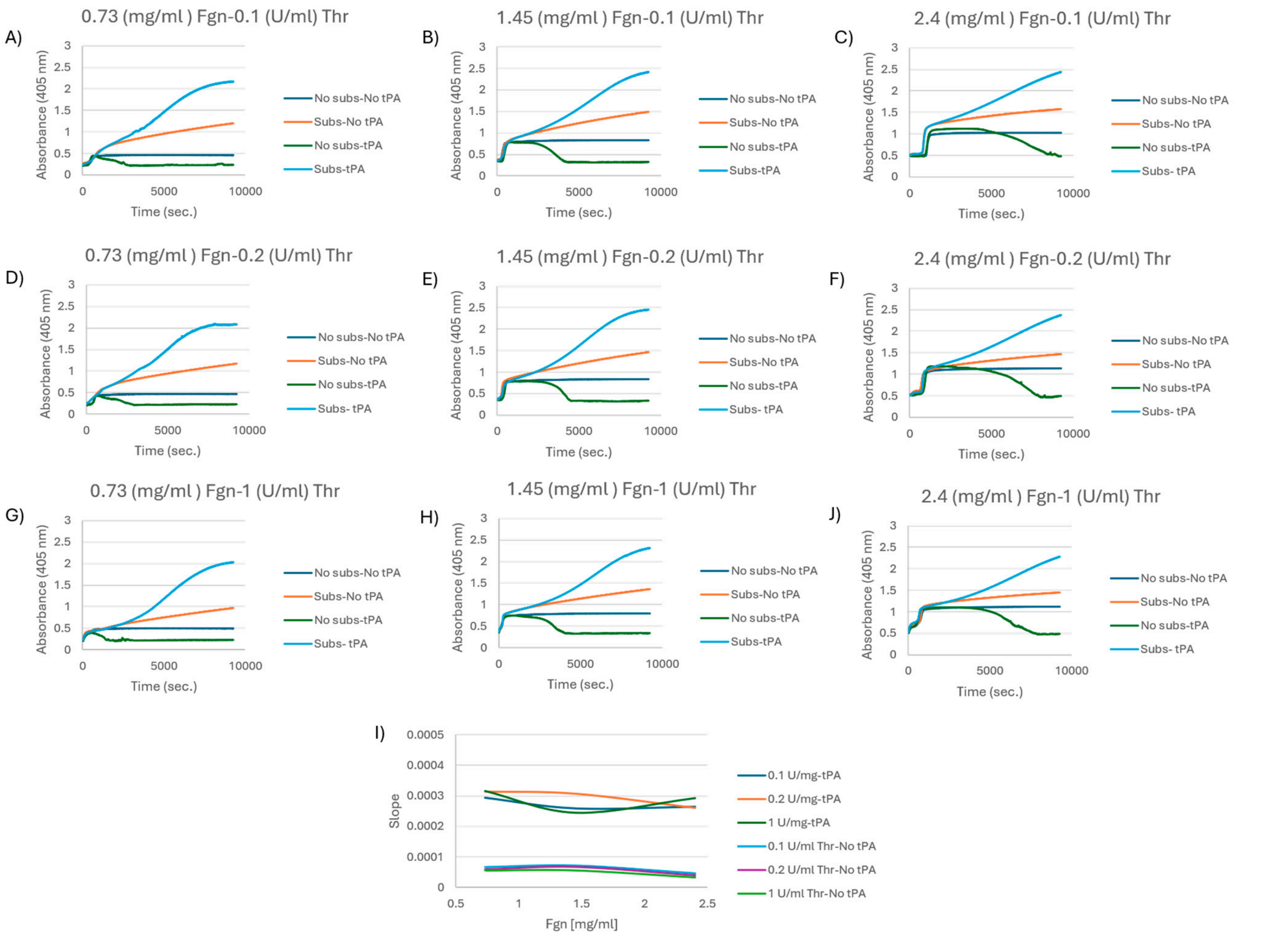

Figure S3. Plasmin generation for different fibrinogen and thrombin concentrations. (A-J) Raw absorbance data for all fibrinogen and thrombin concentrations, with and without added tPA and with and without colorimetric plasmin substrate. (I) The slope (absorption/sec) for all reactions with and without tPA.

S4. AF488-labeled fibrinogen & network formation and structure.

To evaluate whether fluorescent labeling affected fibrin network formation, turbidity measurements were performed using increasing percentages of Alexa Fluorophore 488-labeled (AF488-labeled) fibrinogen (0%, 1.5%, 3%, 6%, and 12% of total fibrinogen). Two independent replicates were analyzed for each condition. Maximum absorbance and clot formation rates were minimally affected between 0% and 6% AF488-labeled fibrinogen, indicating that low labeling percentages did not substantially alter fibrin polymerization or network formation. In contrast, more noticeable differences in maximum absorbance were observed at 12% AF488-labeled fibrinogen, suggesting altered clot structure at higher AF488-labeled fibrinogen concentrations. Additionally, higher percentages of AF488-labeled fibrinogen produced visible aggregates and star-like artifacts during imaging. Based on these results, 6.5% AF488-labeled fibrinogen was selected as the optimal compromise between sufficient fluorescence intensity for imaging and minimal perturbation of fibrin network formation.

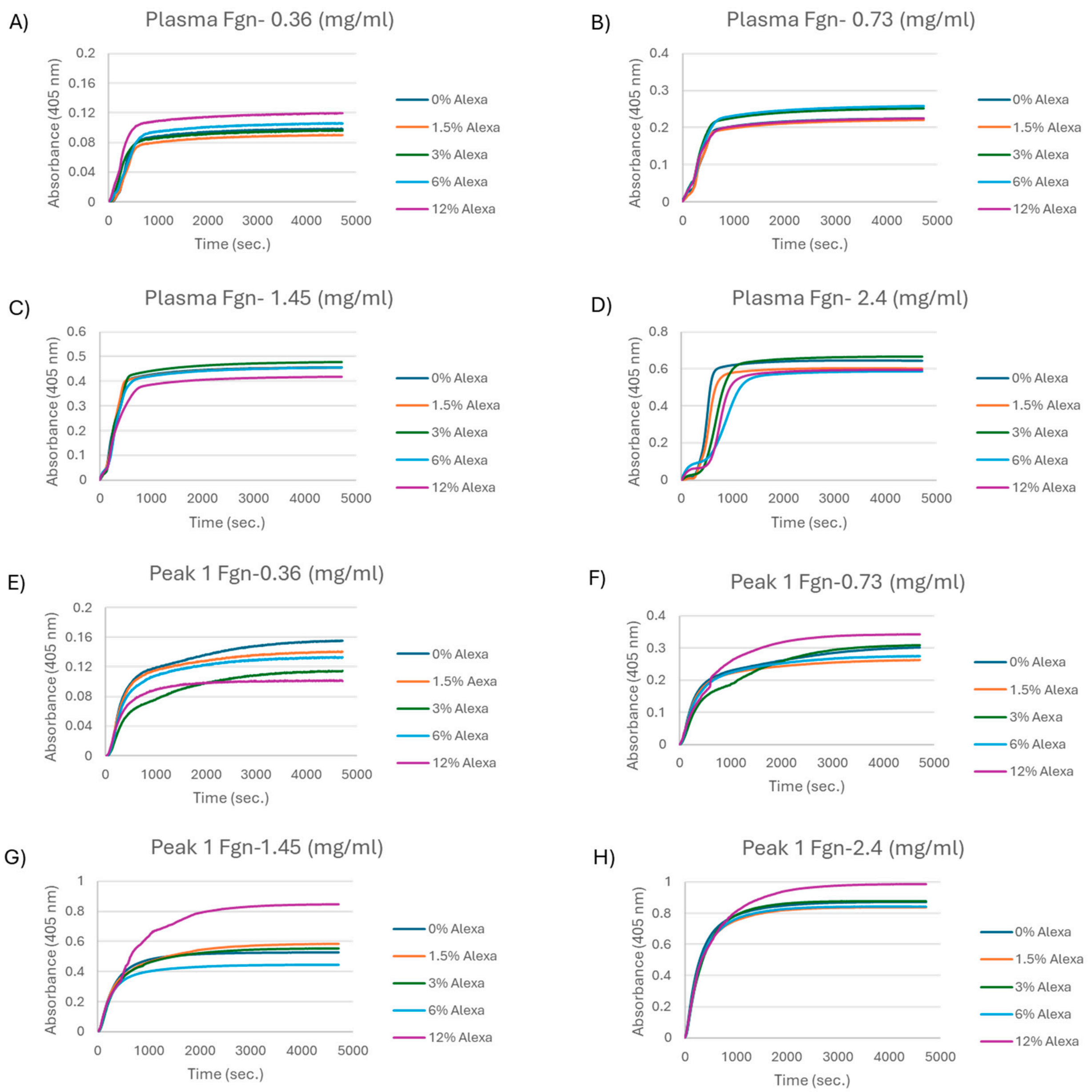

Figure S4. Turbidity curves for samples of different amounts of AF488 labeled fibrinogen added (0%, 1.5%, 3%, 6%, 12%). (A-D) Plasma samples. (E-H) Purified Peak 1 samples. All samples were polymerized using 0.2 U/mL thrombin. Figure is adapted from [26].

S5. Box and whisker plots, and distributions corresponding to Fig. 5A

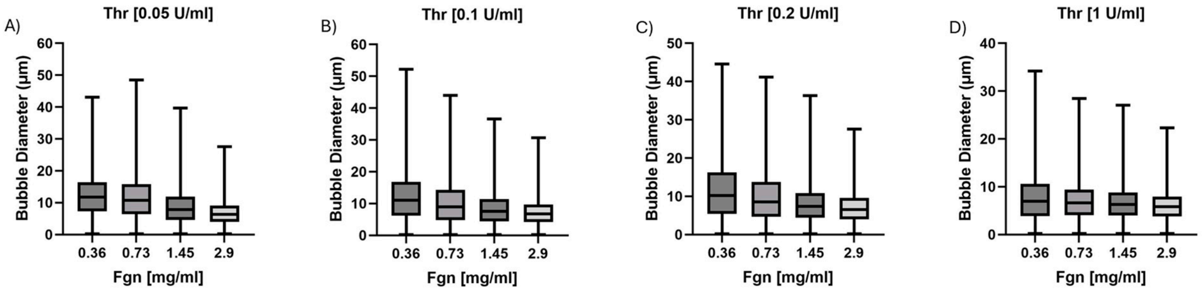

Figure S5. 1: Box and whisker plots of bubble diameter distributions at different fibrinogen and thrombin concentrations (A-D). The center line represents the median bubble diameter, boxes

indicate the interquartile range (25th–75th percentile), and whiskers represent the minimum and maximum values.

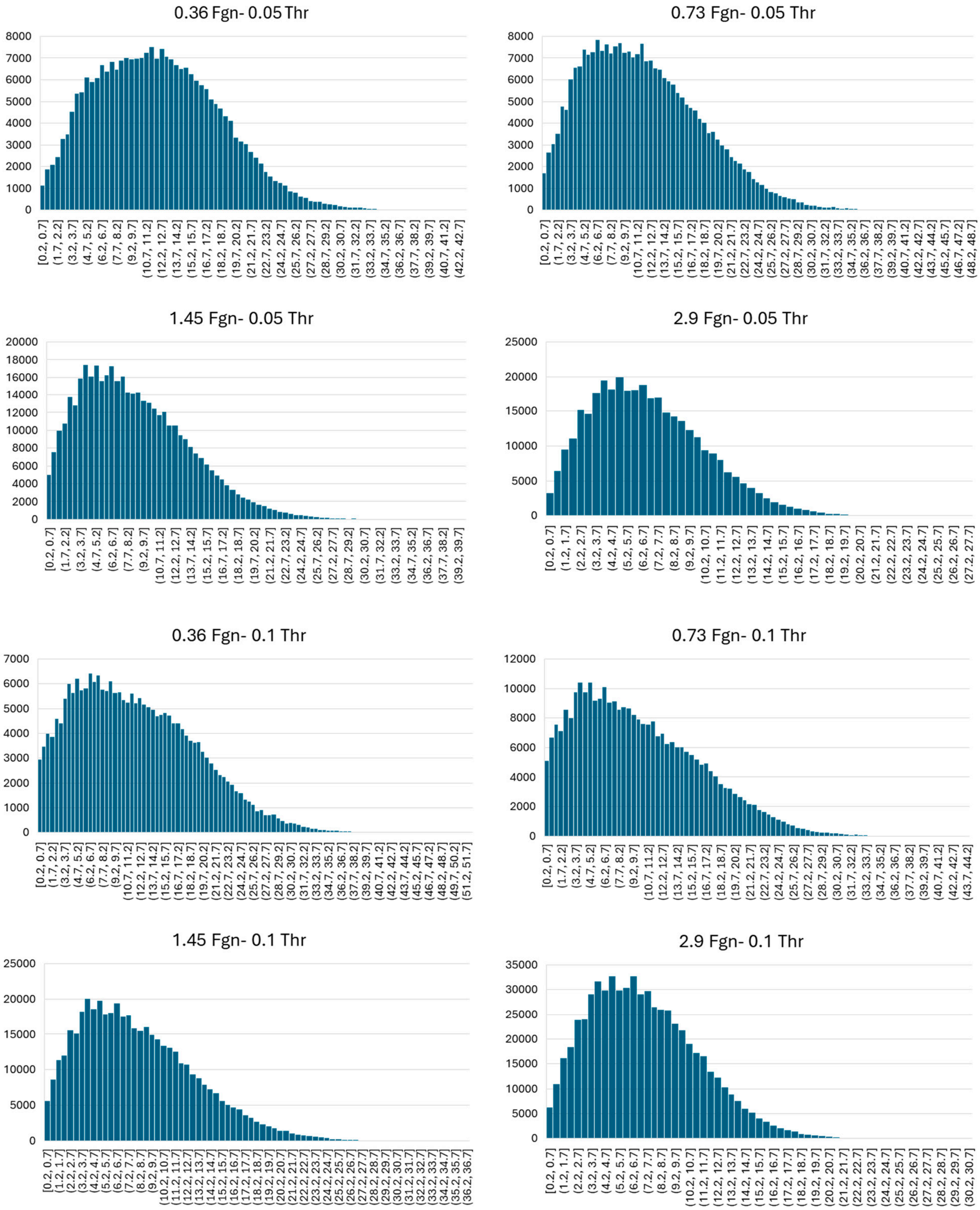

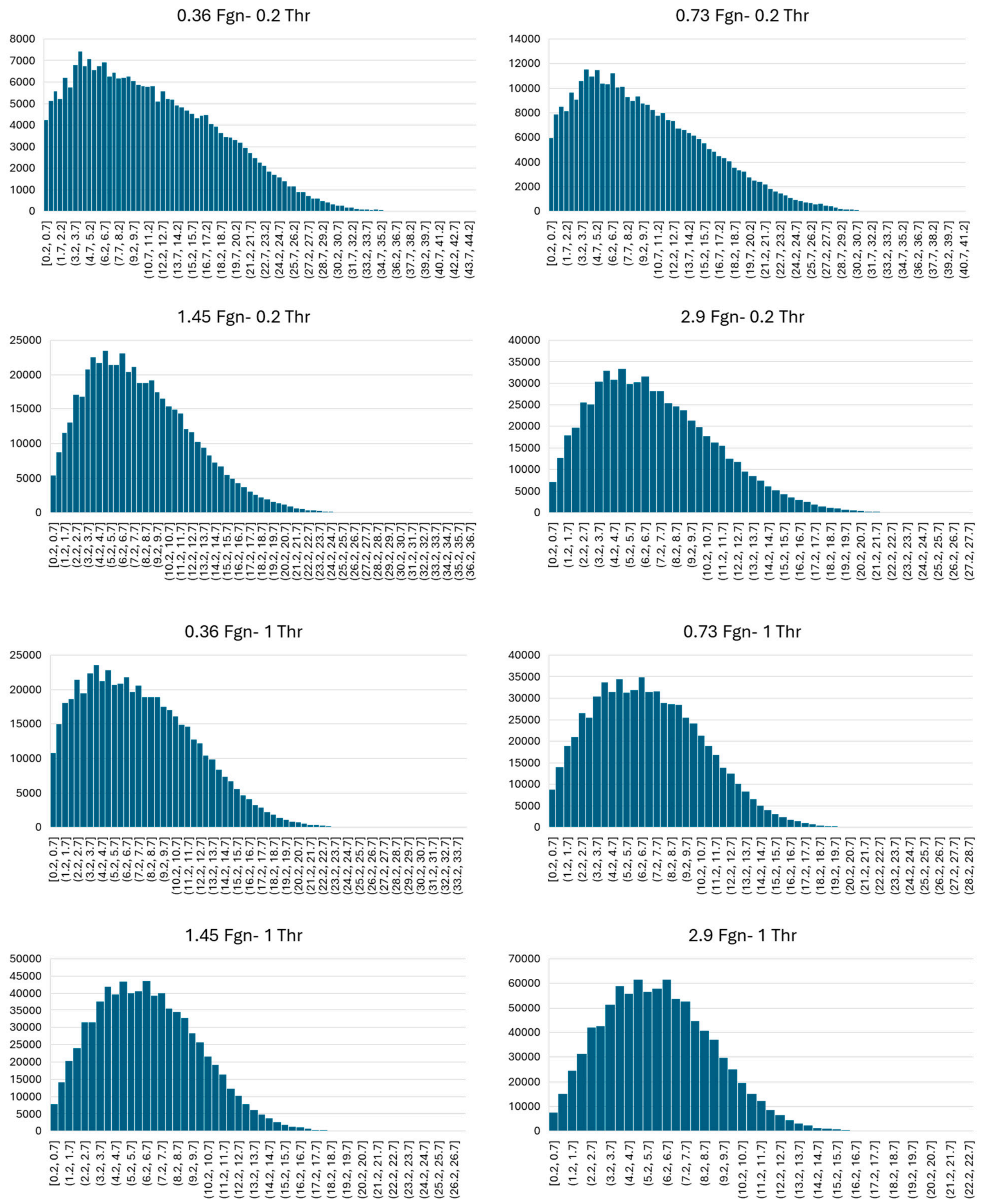

Figure S5. 2: Bubble diameter distributions at varying fibrinogen (mg/mL) and thrombin (U/mL) concentrations. The x-axis represents bubble diameter (μm).

S6. Table S1. Plasma fitting parameters and statistics of bubble diameter (median), and fiber density (mean).

Fibrinogen (mg/mL)-Thrombin (U/mL) concentrations	Bubble diameter Median (IQR)	Fiber density Mean (Mean∓SD)
0.36/0.05	11.75 (7.31-16.44)	6.69∓1.97
0.36/0.1	11.05 (6.16-16.84)	6.54∓0.61

https://doi.org/10.3390/biom16101471

0.36/0.2	10.21 (5.38-16.21)	9.65∓1.19
0.36/1.0	6.96 (3.86-10.57)	21.21∓2.27
0.73/0.05	10.78 (6.43-15.80)	7.11∓1.25
0.73/0.1	8.96 (4.85-14.28)	10.71∓0.73
0.73/0.2	8.55 (4.64-13.71)	12.11∓1.10
0.73/1.0	6.62 (4.08-9.42)	23.71∓2.42
1.45/0.05	7.88 (4.59-11.93)	12.26∓2.56
1.45/0.1	7.52 (4.41-11.40)	13.40∓2.27
1.45/0.2	7.33 (4.46-10.86)	13.99∓1.67
1.45/1.0	6.31 (3.99-8.81)	26.61∓3.31
2.9/0.05	6.96 (4.28-10.17)	18.01∓2.26
2.9/0.1	6.75 (4.18-9.69)	19.87∓4.47
2.9/0.2	6.51 (3.99-9.58)	21.46∓4.05
2.9/1.0	5.84 (3.83-7.94)	31.83∓3.54

S7. Code for Fiber Count Analysis

This is the code run in the macro for imageJ

```
dir1 = getDirectory("Choose Source Directory ");
 format = getFormat();
 dir2 = getDirectory("Choose Destination Directory ");
 list = getFileList(dir1);
 setBatchMode(true);
 for (index=0; index<list.length; index++) {
   showProgress(index+1, list.length);
   open(dir1+list[index]);

        id = getImageID();
        path=File.name;

        run("Z Project...", "projection=[Max Intensity]");
  saveAs("Tiff", dir2+list[index]+3);
        run("Subtract Background...", "rolling=20");
        run("Duplicate...", "title=ImageClot");
        selectImage(id);
        close();
        selectWindow("ImageClot");
        run("Make Binary");

        for (i=0; i<20; i++) {
               newImage("ImageGridV", "8-bit White", 2048, 2048, 1);
               makeLine(51.2 + (i*102.4), 0, 51.2 + (i*102.4), 2048);
               run("Draw");
               run("Select None");
               imageCalculator("Add create 32-bit", "ImageClot","ImageGridV");
               selectWindow("Result of ImageClot");
```

```
                rename(path);
                setThreshold(0, 254);
                run("Make Binary", "thresholded remaining black");
                run("Analyze Particles...", "size=0-Infinity circularity=0.00-1.00 show=Nothing summarize");
                selectWindow("ImageGridV");
                close();
        }

        for (j=0; j<20; j++) {
                newImage("ImageGridH", "8-bit White", 2048, 2048, 1);
                makeLine(0, 51.2 + (j*102.4), 2048, 51.2 + (j*102.4));
                run("Draw");
                run("Select None");
                imageCalculator("Add create 32-bit", "ImageClot","ImageGridH");
                selectWindow("Result of ImageClot");
                rename(path);
                setThreshold(0, 254);
                run("Make Binary", "thresholded remaining black");
                run("Analyze Particles...", "size=0-Infinity circularity=0.00-1.00 show=Nothing summarize");
                selectWindow("ImageGridH");
                close();
        }

        selectWindow("ImageClot");
        close();
        selectWindow("Summary");
        close("\\Others");

        if (format=="8-bit TIFF" || format=="GIF")
     convertTo8Bit();
   /*saveAs(format, dir2+list[index]);*/
   close();
}


 function getFormat() {
    formats = newArray("TIFF", "8-bit TIFF", "JPEG", "GIF", "PNG",
      "PGM", "BMP", "FITS", "Text Image", "ZIP", "Raw");
    Dialog.create("Batch Convert");
    Dialog.addChoice("Convert to: ", formats, "TIFF");
    Dialog.show();
    return Dialog.getChoice();
 }
```

```
 function convertTo8Bit() {
            if (bitDepth==24)
         run("8-bit Color", "number=256");
     else
         run("8-bit");
 }
}
```

S8. Code for Bubble Analysis (Matlab)

```
% Bubble_Analysis_EDM_v2.m
% Keith Bonin - Nov. 10, 2016; March 1, 2022
% Modified by Stephen R. Baker - March 9, 2022
% **There are pauses in this script file.**
% This script uses the EDM method worked out by Munster & Fabry (Biophys.
% J. June 2013) to speed up a 2D bubble analysis of pores in a mesh network
% This script has two dependency functions
% find_bubbles.m
% loadtiff.m
%%----------------------------------------------------------------------
% Clean up
%----------------------------------------------------------------------
clc; % Clear the command window.
close all; % Close all figures (except those of imtool.)
fclose('all'); % Close all open files.
clear all; % Erase all existing variables.
fontSize = 14;
tStart = tic; % Start timer. Pair with tStop = toc;
%%----------------------------------------------------------------------
% This prompts the user first asking if they want to display all of the
% images for each step and then whether they want to save the data.
%----------------------------------------------------------------------
% Ask if the user wants to view the first image in the stack
quest1 = questdlg('Do you want to display all of the images?', ...
'Display Images?', 'Yes', 'No','No');
switch quest1
case 'Yes'
displayFlag = 'yes';
case 'No'
displayFlag = 'no';
otherwise
displayFlag = 'no';
end
% Ask for the save format
quest2 = questdlg(['What format do you want to save the data in?' ...
```

```
newline '*** Close for no Save ***'],...
'Data Format?', '.xlsx', '.csv', '.txt', '.txt');
switch quest2
case '.xlsx'
writeDataToFile = '.xlsx';
case '.csv'
writeDataToFile = '.csv';
case '.txt'
writeDataToFile = '.txt';
otherwise
writeDataToFile = 'no';
end
%displayFlag = 'no';
%writeDataToFile = '.xlsx';
%writeDataToFile = '.csv';
%writeDataToFile = '.txt';
%%------------------------------------------------------------------------
% Read in the data files and set initial variables
%------------------------------------------------------------------------
% Select all of the tif image files you want to analyze
AllFileNames = uigetfile('*.tif*', 'Select All Images (*.tif*)', 'MultiSelect','on');
numImages = numel(AllFileNames); % Determine the total number of files
if class(AllFileNames) == 'char'
numImages = 1;
else
end
%%------------------------------------------------------------------------
% Run through all selected images in the Tiffs folder
%------------------------------------------------------------------------
for fileIndex = 1:numImages
if class(AllFileNames) == 'char'
fileName = [AllFileNames];
else
fileName = [AllFileNames{fileIndex}]; % File name
end
path=[cd,'\Tiffs\']; % Set the path to the folder names Tiffs
imageFile = strcat(path, fileName); % File name with path
bubbleRadiiTotal = []; % Reset the total bubble radii to zero
imageData = loadtiff(imageFile); % Use the loadtiff function to open tiff with multiple planes
[rows, cols, planes] = size(imageData); % planes needed for multitiff files
info = imfinfo(imageFile);
bitDepth = info.BitDepth;
pixPerMicron = info.XResolution;
pixelSize = 1/(pixPerMicron);
```

```
%%------------------------------------------------------------------------
% Run through all slices or planes for each image
%-------------------------------------------------------------------------
for idx = 1:planes
planeData = imread(imageFile,idx); % Read each plane
averagePlaneIntensity = mean(planeData(:)); %Find the average plane intensity
% Check for bit depth. If The image is 16-bit, it will not display correctly
if strcmp(displayFlag, 'yes')
if idx == 1
if bitDepth == 16
maxImageData = max(max(planeData));
imshow(planeData,'DisplayRange',[0 maxImageData]);
else
imshow(planeData,'DisplayRange',[0 255]);
end
pause
disp('hit Enter to continue');
end
end
numPixels = numel(planeData(:,1));
% Remove background noise in image by threshold filtering at 3x the
% average intensity
imageDataBlur = imgaussfilt(planeData,3); % apply Gaussian filter
averageImageIntensity = mean(imageDataBlur(:)); % average intensity
% Set all pixels less than 3x the average intensity to 0
planeData(imageDataBlur < 3*averageImageIntensity) = 0;
binaryImage = planeData;
if strcmp(displayFlag, 'yes')
if idx == 1
imshow(binaryImage); %show the binary image with 3x avg threshold
pause
disp('hit Enter to continue');
end
end
binaryImage(binaryImage>0) = 1; % turn into binary image of 0's and 1's
maxBinary = max(max(binaryImage));
[imageRows imageCols] = size(binaryImage);
%Find the amount of the image covered by fibers
fractionCoverage = sum(binaryImage(:))/(imageRows*imageCols);
% call EMD routine by Munster & Fabry to create bubbles in mesh pores
[bubble_radii bubble_coord] = find_bubbles(binaryImage);
numBubbles = numel(bubble_radii);
bubbleData = zeros(numBubbles,3);
bubbleData(:,1) = bubble_coord(:,1);
```

https://doi.org/10.3390/biom16101471

```
bubbleData(:,2) = bubble_coord(:,2);
bubbleData(:,3) = bubble_radii(:);
binaryImage = 255*binaryImage; % convert 1's to 255's
RGB = repmat(binaryImage,[1,1,3]); % convert the image to an RGB image
maxRGB = max(max(RGB));
%-------------------------------------------------------------------------
% Display fibers and final bubbles.
%-------------------------------------------------------------------------
if strcmp(displayFlag, 'yes')
if idx == 1
imshow(RGB);
axis square;
% Draw bubbles (circles) on image
viscircles([bubbleData(:,2), bubbleData(:,1)], bubbleData(:,3),'EdgeColor','b');
pause
disp('hit Enter to continue');
end
end
%-------------------------------------------------------------------------
% Remove bubbles whose centers are more than 95% away from image edge
%-------------------------------------------------------------------------
removeCount = ones(numBubbles,1);
for k = 1: numBubbles
if (bubble_coord(k,1) < uint16(0.05*numPixels)) || (bubble_coord(k,2) < uint16(0.05*numPixels))...
|| (bubble_coord(k,1) > uint16(0.95*numPixels)) || (bubble_coord(k,2) > uint16(0.95*numPixels))
removeCount(k) = 0;
end
end
numCount = nnz(removeCount);
bubbleDataNew = zeros(numCount,3);
count = 1;
for k = 1: numBubbles
if removeCount(k) == 1;
bubbleDataNew(count,:) = [bubble_coord(k,1) bubble_coord(k,2) bubble_radii(k)];
count = count + 1;
end
end
%-------------------------------------------------------------------------
% Display fibers and final bubbles.
%-------------------------------------------------------------------------
if strcmp(displayFlag, 'yes')
if idx == 1
imshow(RGB);
axis square;
```

```
% Draw bubbles (circles) on image
viscircles([bubbleDataNew(:,2), bubbleDataNew(:,1)], bubbleDataNew(:,3),'EdgeColor','b');
pause
disp('hit Enter to continue');
end
end
%------------------------------------------------------------------------
% Sort bubbles from largest to smallest
%------------------------------------------------------------------------
[Y,I]=sort(bubbleDataNew(:,3),'descend');
bubbleDataNewSorted = bubbleDataNew(I,:); %use the column indices from sort() to sort all columns of A.
bubble_coord = bubbleDataNewSorted(:,1:2);
bubble_radii = bubbleDataNewSorted(:,3);
%------------------------------------------------------------------------
% Remove smaller bubbles that are 'inside' larger bubbles.
% This means that if the center of the smaller bubble is inside the larger
% bubble then we remove the smaller bubble from the list.
%------------------------------------------------------------------------
numBubbles = numCount;
removeCount = ones(numBubbles,1);
for k = 1: numBubbles-1
refyLarge = bubble_coord(k,1);
refxLarge = bubble_coord(k,2);
for kk = k+1: numBubbles
refySmaller = bubble_coord(kk,1);
refxSmaller = bubble_coord(kk,2);
separation = sqrt((refyLarge - refySmaller).^2 + (refxLarge - refxSmaller).^2);
if separation < bubble_radii(k)
removeCount(kk) = 0;
end
end
end
numCount = nnz(removeCount);
bubbleDataFinal = zeros(numCount,3);
count = 1;
for k = 1: numBubbles
if removeCount(k) == 1;
bubbleDataFinal(count,:) = [bubble_coord(k,1) bubble_coord(k,2) bubble_radii(k)];
count = count + 1;
end
end
%------------------------------------------------------------------------
% Display fibers and final bubbles.
%------------------------------------------------------------------------
```

https://doi.org/10.3390/biom16101471

```
if strcmp(displayFlag, 'yes')
if idx == 1
imshow(RGB);
axis square;
% Draw bubbles (circles) on image
viscircles([bubbleDataFinal(:,2), bubbleDataFinal(:,1)], bubbleDataFinal(:,3),'EdgeColor','b');
pause
end
end
%------------------------------------------------------------------------
% Find the bubble radii.
%------------------------------------------------------------------------
bubble_radii = bubbleDataFinal(:,3);
if idx == 1
bubbleRadiiTotal = bubble_radii;
else
bubbleRadiiTotal = [bubbleRadiiTotal; bubble_radii];
end
%------------------------------------------------------------------------
% Find probability density of bubble radii.
%------------------------------------------------------------------------
fillingRatio = pi*sum(bubble_radii.*bubble_radii)/(imageRows*imageCols);
averageRadius = mean(bubble_radii);
bubbleDiameterTotal = 2*bubbleRadiiTotal*pixelSize; % multiply the radius by 2 and then microns/pixels to get the diameter in microns
%h = histogram(bubbleDiameterTotal,10, 'Normalization', 'pdf');
end
figure
h = histogram(bubbleDiameterTotal, 'Normalization', 'pdf');
title([fileName]);
xlabel('Bubble Diameter (\mum)');
ylabel('Normalaized Distribution (pdf)');
% **********************************************************************
% Save bubble the diameters for each image to an file
% **********************************************************************
mkdir('Distributions'); % Make a new folder to store the excel files
path = [cd,'\Distributions\'];
[filepath, name, ext]= fileparts(fileName);
NewFileName = name;
switch writeDataToFile
case '.xlsx'
xlsFilenameOutput = [NewFileName '_Bubble_Diameters.xlsx'];
xlsFileOutput = strcat(path, xlsFilenameOutput);
%create the cell array containing the column header
```

```
columnHeader = {'Bubble Diameters (um)'};
%write the column headers first
xlswrite(xlsFileOutput, columnHeader);
%write the data directly underneath the column headers
lastRow = num2str(numel(bubbleDiameterTotal)+1);
Range = ['A2:A' lastRow];
xlswrite(xlsFileOutput, bubbleDiameterTotal,'Sheet1',Range);
case '.csv'
csvFilenameOutput = [NewFileName '_Bubble_Diameters.csv'];
csvFileOutput = strcat(path, csvFilenameOutput);
%create the cell array containing the column header
columnHeader = {'Bubble Diameters (um)'};
T = table(bubbleDiameterTotal, 'VariableNames',columnHeader);
writetable(T, csvFileOutput);
case '.txt'
txtFilenameOutput = [NewFileName '_Bubble_Diameters.txt'];
txtFileOutput = strcat(path, txtFilenameOutput);
%create the cell array containing the column header
columnHeader = {'Bubble Diameters (um)'};
T = table(bubbleDiameterTotal, 'VariableNames',columnHeader);
writetable(T, txtFileOutput);
end
end
tEnd = toc(tStart) % pair 2: toc
```

Find bubbles code:

```
function [bubble_radii bubble_coord]=find_bubbles(img)
% find_bubbles determines the location and radii of 2D or 3D bubbles in
% the binary image img.
%
% 19|03|2013 Stefan Münster
EDM=bwdist(img); % This calculation of the EDM of the fluid phase assumes
% that fluid pixels are 0 and solid pixels are 1;
% bwdist computes the Euclidean distance transform of the
% binary image img. For each pixel in img, the distance
% transform assigns a number that is the distance between
% that pixel and the nearest nonzero pixel of img.
smoothed_EDM=imfilter(EDM,fspecial('gaussian',5,1)); % this smoothing
% suppresses bubbles of similar size in close proximity and can be omitted
local_maxima=imregionalmax(smoothed_EDM); % determines the local maxima,
% which are the center locations of the bubbles
if(length(size(img))<3) % translates local maxima into x,y,z coordinates
[bubble_coord(:,1) bubble_coord(:,2)]=ind2sub(size(img),find(local_maxima));
else
[bubble_coord(:,1) bubble_coord(:,2) bubble_coord(:,3)]=ind2sub(size(img),find(local_maxima));
```

```
end
bubble_radii=EDM(local_maxima); % determines the radii of the bubbles from
% the EDM values at the local maxima
End
```

Loadtiff Code

```
% Copyright (c) 2012, YoonOh Tak
% All rights reserved.
%
% Redistribution and use in source and binary forms, with or without
% modification, are permitted provided that the following conditions are
% met:
%
% * Redistributions of source code must retain the above copyright
% notice, this list of conditions and the following disclaimer.
% * Redistributions in binary form must reproduce the above copyright
% notice, this list of conditions and the following disclaimer in
% the documentation and/or other materials provided with the distribution
% * Neither the name of the Gwangju Institute of Science and Technology (GIST), Republic of Korea nor the names
% of its contributors may be used to endorse or promote products derived
% from this software without specific prior written permission.
%
% THIS SOFTWARE IS PROVIDED BY THE COPYRIGHT HOLDERS AND CONTRIBUTORS "AS IS"
% AND ANY EXPRESS OR IMPLIED WARRANTIES, INCLUDING, BUT NOT LIMITED TO, THE
% IMPLIED WARRANTIES OF MERCHANTABILITY AND FITNESS FOR A PARTICULAR PURPOSE
% ARE DISCLAIMED. IN NO EVENT SHALL THE COPYRIGHT OWNER OR CONTRIBUTORS BE
% LIABLE FOR ANY DIRECT, INDIRECT, INCIDENTAL, SPECIAL, EXEMPLARY, OR
% CONSEQUENTIAL DAMAGES (INCLUDING, BUT NOT LIMITED TO, PROCUREMENT OF
% SUBSTITUTE GOODS OR SERVICES; LOSS OF USE, DATA, OR PROFITS; OR BUSINESS
% INTERRUPTION) HOWEVER CAUSED AND ON ANY THEORY OF LIABILITY, WHETHER IN
% CONTRACT, STRICT LIABILITY, OR TORT (INCLUDING NEGLIGENCE OR OTHERWISE)
% ARISING IN ANY WAY OUT OF THE USE OF THIS SOFTWARE, EVEN IF ADVISED OF THE
% POSSIBILITY OF SUCH DAMAGE.
tStart = tic;
warn_old = warning('off', 'all'); % To ignore unknown TIFF tag.
%% Check directory and file existence
path_parent = pwd;
[pathstr, ~, ~] = fileparts(path);
if ~isempty(pathstr) && ~exist(pathstr, 'dir')
error 'Directory is not exist.';
end
if ~exist(path, 'file')
error 'File is not exist.';
end
%% Open file
```

https://doi.org/10.3390/biom16101471

```
file_opening_error_count = 0;
while ~exist('tiff', 'var')
try
tiff = Tiff(path, 'r');
catch
file_opening_error_count = file_opening_error_count + 1;
pause(0.1);
if file_opening_error_count > 5 % automatically retry to open for 5 times.
reply = input('Failed to open the file. Do you wish to retry? Y/n: ', 's');
if isempty(reply) || any(upper(reply) == 'Y')
file_opening_error_count = 0;
else
error(['Failed to open the file "' path '".']);
end
end
end
end
%% Load image information
tfl = 0; % Total frame length
tcl = 1; % Total cell length
while true
try
tfl = tfl + 1; % Increase frame count
iinfo(tfl).w = tiff.getTag('ImageWidth');
iinfo(tfl).h = tiff.getTag('ImageLength');
iinfo(tfl).spp = tiff.getTag('SamplesPerPixel');
iinfo(tfl).bps = tiff.getTag('BitsPerSample');
iinfo(tfl).color = iinfo(tfl).spp > 2; % Grayscale: 1(real number) or 2(complex number), Color: 3(rgb), 4(rgba), 6(rgb, complex number), or 8(rgba, complex number)
iinfo(tfl).complex = any(iinfo(tfl).spp == [2 6 8]);
if tiff.getTag('Photometric') == Tiff.Photometric.Palette
disp('Warning: Palette information has been removed.');
end
catch
error 'Failed to load image';
end
%% Sample format
switch tiff.getTag('SampleFormat')
% Unsupported Matlab data type: char, logical, cell, struct, function_handle, class.
case Tiff.SampleFormat.UInt
switch tiff.getTag('BitsPerSample')
case 8
iinfo(tfl).sf = 'uint8';
case 16
```

```
iinfo(tfl).sf = 'uint16';
case 32
iinfo(tfl).sf = 'uint32';
end
case Tiff.SampleFormat.Int
switch tiff.getTag('BitsPerSample')
case 8
iinfo(tfl).sf = 'int8';
case 16
iinfo(tfl).sf = 'int16';
case 32
iinfo(tfl).sf = 'int32';
end
case Tiff.SampleFormat.IEEEFP
switch tiff.getTag('BitsPerSample')
case 32
iinfo(tfl).sf = 'single';
case 64
iinfo(tfl).sf = 'double';
end
otherwise
% (Unsupported)Void, ComplexInt, ComplexIEEEFP
error 'Unsupported Matlab data type. (char, logical, cell, struct, function_handle, class)';
end
if tfl > 1
% If tag information is changed, make a new cell
if iinfo(tfl-1).w ~= iinfo(tfl).w || ...
iinfo(tfl-1).h ~= iinfo(tfl).h || ...
iinfo(tfl-1).spp ~= iinfo(tfl).spp || ...
any(iinfo(tfl-1).sf ~= iinfo(tfl).sf) || ...
iinfo(tfl-1).color ~= iinfo(tfl).color || ...
iinfo(tfl-1).complex ~= iinfo(tfl).complex
tcl = tcl + 1; % Increase cell count
iinfo(tfl).fid = 1; % First frame of this cell
else
iinfo(tfl).fid = iinfo(tfl-1).fid + 1;
end
else
iinfo(tfl).fid = 1; % Very first frame of this file
end
iinfo(tfl).cid = tcl; % Cell number of this frame
if tiff.lastDirectory(), break; end;
tiff.nextDirectory();
end
```

```
%% Load image data
if tcl == 1 % simple image (no cell)
if ~iinfo(tfl).color
oimg = zeros(iinfo(tfl).h,iinfo(tfl).w,iinfo(tfl).fid, iinfo(tfl).sf); % Grayscale image
else
oimg = zeros(iinfo(tfl).h,iinfo(tfl).w,iinfo(tfl).spp,iinfo(tfl).fid, iinfo(tfl).sf); % Color image
end
for tfl = 1:tfl
tiff.setDirectory(tfl);
temp = tiff.read();
if iinfo(tfl).complex
temp = temp(:,:,1:2:end-1,:) + temp(:,:,2:2:end,:)*1i;
end
if ~iinfo(tfl).color
oimg(:,:,iinfo(tfl).fid) = temp; % Grayscale image
else
oimg(:,:,:,iinfo(tfl).fid) = temp; % Color image
end
end
else % multiple image (multiple cell)
oimg = cell(tcl, 1);
for tfl = 1:tfl
tiff.setDirectory(tfl);
temp = tiff.read();
if iinfo(tfl).complex
temp = temp(:,:,1:2:end-1,:) + temp(:,:,2:2:end,:)*1i;
end
if ~iinfo(tfl).color
oimg{iinfo(tfl).cid}(:,:,iinfo(tfl).fid) = temp; % Grayscale image
else
oimg{iinfo(tfl).cid}(:,:,:,iinfo(tfl).fid) = temp; % Color image
end
end
end
%% Close file
tiff.close();
cd(path_parent);
warning(warn_old);
display(sprintf('The file was loaded successfully. Elapsed time : %.3f s.', toc(tStart)));
end
```

https://doi.org/10.3390/biom16101471
